# No differences in neural responses or performance during cannabis cue-specific inhibitory control tasks between recreational cannabis users and non-users: Insights from fNIRS

**DOI:** 10.1177/02698811251358814

**Published:** 2025-10-02

**Authors:** Christopher R. Pickering, Valentina Lorenzetti, Andrew Jones, Martin Guest, Paul Christiansen, Carl A. Roberts

**Affiliations:** 1 Department of Psychology, University of Liverpool, UK; 2 Faculty of Health Sciences, Neuroscience of Addiction and Mental Health Program, Healthy Brain and Mind Research Centre, School of Health and Behavioural Sciences, Australian Catholic University, Fitzroy, Australia; 3 Faculty of Health, School of Psychology, Liverpool John Moores University, UK

**Keywords:** cannabis, inhibitory control, addiction, fNIRS, neuroimaging

## Abstract

**Background:**

Impaired inhibitory control has been observed in regular cannabis users. Theories suggest that regular cannabis use is maintained by reward-driven behaviour, which may be underpinned by adaptations in neural reward and inhibitory control systems, thus increasing vulnerability to dependency.

**Aims:**

This study investigated neural correlates of cannabis cue-specific inhibitory control in regular cannabis users and non-users using functional near-infrared spectroscopy (fNIRS).

**Methods:**

Thirty regular cannabis users and thirty non-user controls completed two inhibitory control tasks (Go/No/Go and Stop-Signal Task), and a measure of attentional bias (Cannabis Stroop task). fNIRS recorded prefrontal and orbitofrontal haemodynamic responses (oxygenated haemoglobin and deoxygenated haemoglobin). Group comparisons and exploratory regressions examined cannabis use characteristics as predictors of behavioural and neural outcomes.

**Results:**

No significant group differences were found in behavioural performance or haemodynamic activity across tasks. Exploratory regressions showed no significant associations between cannabis use characteristics and behavioural or neural outcomes after adjusting for covariates.

**Conclusions:**

No evidence of impaired inhibitory control, attentional bias, or differences in prefrontal function were found in non-dependent cannabis users. Future studies should investigate whether such deficits emerge with heavier or dependent use.

## Introduction

Cannabis is one of the most widely used psychoactive substances globally, with an estimated 228 million users in 2022, representing over 4% of the global adult population (United Nations Office on Drugs and Crime, 2023). Although the risks of harm associated with cannabis are generally considered lower than for substances such as alcohol or opioids (Nutt et al., 2010), regular use is associated with cognitive impairments, psychosocial problems and increased risk of developing cannabis use disorder (CUD), particularly among daily users (Connor et al., 2021; Solmi et al., 2023). These risks are exacerbated by rising Δ9-tetrahydrocannabinol (THC) potency (ElSohly et al., 2021) and decreasing public concern about the harms of use (Harrison et al., 2023; Waddell, 2022).

The primary psychoactive compound in cannabis, THC, activates CB^1^ receptors, which are densely distributed in brain regions critical to executive function, emotional regulation and reward processing (Mackie, 2005). Regular cannabis use has been associated with neuroadaptations in these areas, particularly in the prefrontal cortex (PFC), anterior cingulate cortex (ACC) and orbitofrontal cortex (OFC; Gilman et al., 2014; Lorenzetti et al., 2019; Zhou et al., 2018). These changes may contribute to cognitive impairments, including deficits in attention, working memory, processing speed and inhibitory control (Crean et al., 2011; Lovell et al., 2020).

Inhibitory control is a core component of addiction vulnerability and maintenance (Luijten et al., 2014; Yücel et al., 2019) and is often studied using behavioural paradigms such as the Go/No-Go task (GNG) and the Stop-Signal Task (SST; Logan and Cowen, 1984). Evidence suggests cannabis users may exhibit poorer performance on the SST, while findings for the GNG are less consistent (Liu et al., 2019). Notably, some functional Magnetic Resolution Imaging (fMRI) studies have identified functional differences in cannabis users compared to controls - such as ACC hypoactivity and increased PFC activation - despite comparable task performance, possibly indicating compensatory neural activity (Hester et al., 2009; Tapert et al., 2007). These tasks reflect distinct subcomponents of motor inhibition: action restraint and action cancellation (Schachar et al., 2007). The GNG captures restraint (i.e., preventing a response before it is initiated) while the SST assesses cancellation (i.e., interrupting an ongoing motor response). Although both forms of inhibition are relevant to addiction, they may rely on partially dissociable neural substrates and show differential sensitivity to cannabis-related impairments.

Standard inhibitory control tasks typically use neutral stimuli, which may not fully reflect the influence of drug-related attentional bias. Cannabis users demonstrate increased neural and behavioural reactivity to cannabis-related cues (O’Neill et al., 2020; Sehl et al., 2021), and such cue-reactivity is implicated in craving and relapse (Berridge and Robinson, 2016; Zilverstand et al., 2018). Cannabis-related versions of inhibitory control tasks can help assess whether attentional biases and reward signalling impair inhibitory control (Field and Cox, 2008).

This study used functional near-infrared spectroscopy (fNIRS), a non-invasive imaging method suitable for measuring cortical haemodynamics, to assess PFC and OFC activity during two inhibitory control tasks (GNG and SST), and a measure of attentional bias (Cannabis Stroop task), each incorporating cannabis-related stimuli.

It was hypothesised that cannabis users, relative to non-user controls, would show reduced task performance and differences in neural activity – specifically differences in PFC activity during inhibition and OFC activity in response to cannabis cues.

## Method

### Participants

A total of 60 participants (30 cannabis users, 30 controls) were recruited via advertisements on the University of Liverpool campus. The cannabis user group included 18 females and 12 males (*M*_age_ = 21.30, SD = 2.81), and the control group included 20 females and 10 males (*M*_age_ = 21.33, SD = 3.22). There were no significant group differences in age or sex distribution. Ethical approval was granted by the University of Liverpool Research Ethics Committee (Ref: 10754).

#### Inclusion and exclusion criteria

Cannabis users reported using cannabis at least once weekly for the past 3 months (Borissova et al., 2022); controls had used cannabis fewer than five times. All the participants were aged 18–30, with no history of psychiatric, neurological or substance use disorders, and no current use of psychoactive medication. The age range was intended to minimise age-related variability in executive function, which develops into the mid-20s and may begin to decline from the late 20s (Hartshorne and Germine, 2015; Salthouse, 2009). Substance use history beyond cannabis was not restricted, to reflect typical patterns among cannabis users (Rosen et al., 2018). Participants were required to not have used cannabis on the day of the study.

#### Design

The study used a between-groups design, comparing cannabis users and controls on task performance (i.e. accuracy, reaction time) and prefrontal haemodynamic responses (i.e. oxygenated haemoglobin (oxyHb), deoxygenated haemoglobin (deoxyHb)) across three inhibitory control tasks.

### Materials

#### Questionnaires

Participants completed a battery of self-report measures, including the Background Drug Use Questionnaire (Montgomery et al., 2005) to assess current and lifetime substance use, including estimated cannabis consumption in Standard Joint Units (SJUs; Kögel et al., 2017). Additional measures included the Mood Adjective Checklist (MAC; Fisk and Warr, 1996) to assess anxiety, arousal and hedonic tone; the National Aeronautics and Space Administration - Task Load Index (NASA-TLX; Hart and Staveland, 1988) to evaluate perceived cognitive workload (excluding physical demand); Raven’s Progressive Matrices (RPM; Penrose and Raven, 1936) to assess non-verbal fluid intelligence; and the Alcohol Use Disorders Identification Test - Consumption (AUDIT-C; Bush et al., 1998) for alcohol use.

### Behavioural tasks

#### GNG

This task measured action restraint, a subcomponent of inhibitory control, using cannabis-related images (Macatee et al., 2021), adapted from Houben and Jansen (2011). Participants were instructed to respond to landscape-oriented images (Go trials) and withhold responses to portrait-oriented images (No-Go trials). The task consisted of 160 trials, with a Go:No-Go ratio of 3:1 (75% Go), following a brief practice phase. The primary behavioural outcome was the number of commission errors on No-Go trials (Logan and Cowan, 1984).

#### Cannabis Stroop task

This modified emotional Stroop assessed attentional bias. Participants identified the font colour of cannabis-related or neutral words while ignoring word content. Stroop interference was calculated as the difference in response times between cannabis and neutral words. Stimuli were matched for word length and syllables and counterbalanced across participants (Field, 2005).

#### SST

This task measured action cancellation, the ability to inhibit a response already in progress. It used cannabis-related visual stimuli (Macatee et al., 2021) and required participants to make rapid motor responses to image orientation unless a stop-signal (‘=’) appeared shortly after the Go signal, indicating they should inhibit their response. The stop-signal delay was dynamically adjusted using a staircase procedure. The task included 160 trials (120 Go, 40 Stop-Signal). The primary outcome was Stop Signal Reaction Time (SSRT), computed using the integration method (Verbruggen et al., 2019), and reflects the unobserved latency to inhibit behaviour in milliseconds (Verbruggen and Logan, 2008).

Detailed protocols and materials for all tasks are provided in Appendix A.

#### fNIRS data collection and pre-processing

fNIRS data was recorded using an 18-channel NTS2 continuous-wave fNIRS system (Gowerlabs Ltd., n.d., UK) during task performance. The optode array targeted regions of the PFC involved in inhibitory control and reward processing. Data were pre-processed in Homer3 (Huppert et al., 2009) using motion correction, band-pass filtering and standard conversion methods. Full configuration, pre-processing pipeline and exclusion criteria are detailed in Appendix A.

### Procedure

Participants attended a single 2-hour lab session at the University of Liverpool’s Brain and Behaviour Laboratory. After providing informed consent, participants completed the Background Drug Use Questionnaire, AUDIT-C, MAC and RPM. The fNIRS cap was fitted, and a 10-seconds resting baseline was recorded prior to task onset, during a 2-minute period in which participants viewed neutral ocean footage to minimise arousal. Baseline recordings were repeated before each task.

**Figure 1.**
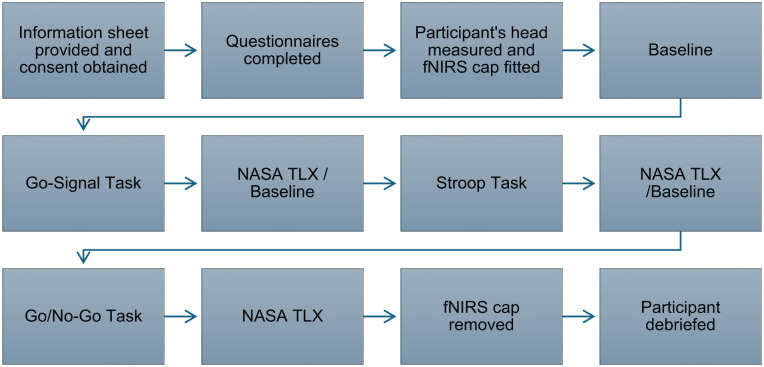
Flowchart of experimental procedure.

Participants completed the GNG, Cannabis Stroop and SSTs in a fixed order while fNIRS data were collected. After tasks, they completed the NASA-TLX to assess cognitive workload. Upon completion, participants were debriefed and compensated with a £20 shopping voucher. See (Figure 1) for a flowchart of the experimental procedure.

#### Statistical analysis

Group differences in task performance - GNG errors (commission errors reflecting failures of action restraint), Stroop interference reaction time (in milliseconds, indicating attentional bias and impaired interference control), and SSRT (the unobserved latency to inhibit an already initiated response), as well as subjective cognitive demand (NASA-TLX), were assessed using independent samples *t*-tests.

For the fNIRS data, changes in oxyHb and deoxyHb concentrations from baseline were analysed separately using univariate analyses of variance (ANOVAs) at each of the 18 channels, with group (cannabis users vs controls) as the between-subjects factor. A partial correction threshold of *p* < 0.01 was applied to adjust for multiple comparisons.

Exploratory hierarchical linear regressions were also conducted within the cannabis user group to assess whether individual differences in use characteristics (e.g. lifetime dose, age of onset, 30-day frequency) predicted behavioural performance and fNIRS activation, adjusting for age, sex and alcohol use (AUDIT-C). These models were designed to explore individual variation in neural and behavioural outcomes as a function of use severity, including potential patterns of compensatory activation versus disengagement or dose-dependent effects.

## Results

### Baseline characteristics and group comparisons

Baseline indices of fluid intelligence, mood and alcohol use are summarised in Table 1.

**Table 1.** Baseline indices of fluid intelligence, mood and alcohol use.

Measure	Cannabis users	Controls	*t*-Value	*df*	*p*-Value	*d*-Value
RPM (maximum 24)	18.57 (±3.17)	20.03 (±2.21)	2.08	58	0.042	0.54
MAC anxiety	11.20 (±2.14)	12.31 (±2.29)	1.93	57	0.059	0.50
MAC arousal	21.80 (±3.03)	21.31 (±2.63)	−0.66	57	0.511	−0.17
MAC hedonic tone	24.60 (±2.92)	24.03 (±2.65)	−0.78	57	0.440	−0.20
AUDIT-C	6.57 (±2.64)	5.23 (±2.92)	−1.86	58	0.068	−0.48

Due to a procedural error, MAC (Fisk and Warr, 1996) data was not collected for one participant.

RPM: Raven’s progressive matrices; MAC: mood adjective checklist; AUDIT-C: alcohol use disorders identification test – consumption.

Independent samples *t*-tests revealed that controls scored significantly higher than cannabis users on RPM. No group differences were found in state anxiety, arousal, hedonic tone (MAC) or alcohol use (AUDIT-C). Both groups had average AUDIT-C scores above the clinical cut-off for hazardous drinking (⩾5).

Self-reported abstinence durations (days since last use) were collected for cannabis and other substances, with participants reporting up to a maximum of 10 days. Among cannabis users, mean abstinence from cannabis was 2.08 days (SD = 1.52). Alcohol abstinence was also reported, with cannabis users reporting a mean of 3.50 days (SD = 2.38) and controls reporting a mean of 3.33 days (SD = 2.09). Abstinence data for other substances (e.g. 3,4-methylenedioxymethamphetamine [MDMA], ketamine and cocaine) are presented in Appendix B.

#### Summary of recent drug use by group

Table 3 presents the number of participants in each group who reported substance use within the past 3 months. These figures are based on self-reported usage frequencies and are intended to contextualise the behavioural and neurophysiological findings. For clarity of reporting, frequency categories (‘Less than monthly’, ‘At least once per month,’ ‘At least once per week’ and ‘Daily’) were collapsed into a single classification (‘Used in the last 3 months’). Comprehensive frequency distributions, including reports of substances ‘used previously but not within the past 3 months’, are provided in Appendix B.

As shown in Tables 2 and 3, participants in the cannabis group reported extensive cannabis use and a high prevalence of other substance use, indicating a polydrug user profile. Notably, seven participants in the control group reported lifetime cannabis use (range = 1–4 SJUs), and limited polydrug use was also observed among controls. In addition to abstinence and frequency data, participants estimated their total consumption of each substance over the past 30 days. These quantities are presented in Appendix B.

**Table 2.** Indices of cannabis and lifetime dose of other substances.

	Cannabis users	
Substance use indices	Mean (±SD)	Control
Indices of cannabis use		
Age of onset (years)	16.03 (±2.47)	
Last 30 days (SJUs)	41.33 (±43.05)	
Last 30 days (frequency)	16.73 (±10.88)	
Lifetime dose (total SJUs)	3243.89 (±8490.12)	0.53 (±6.57), *N* = 4
Lifetime doses		
Cocaine (grams)	24.86 (±35.57), *N* = 15	0.60 (±.35), *N* = 3
Ecstasy (MDMA; milligrams)	100064.00 (±27668.71), *N* = 15	150, *N* = 1
Ketamine (grams)	26.99 (±27.38), *N* = 16	12.60 (±20.27), *N* = 3
LSD (micrograms)	5369.00 (±8726.07), *N* = 5	

As not all participants reported use of other substances, *N*’s vary by variable and are reported within the table.

**Table 3.** Frequencies of recent drug use (within last 3 months) by group.

Substance	Cannabis users	Control
Alcohol	30	26
Amphetamine	1	0
Cocaine	13	2
Ecstasy (MDMA)	8	0
Ketamine	18	2
LSD	2	0
Mushrooms (psilocybin)	3	0
Poppers (alkyl nitrites)	8	0
Tobacco	22	8

Tobacco use was also examined given its reported cognitive effects (Valentine and Sofuorgo, 2018). Twenty-two cannabis users and eight controls reported tobacco use over the last 3 months, with daily use reported by ten cannabis users and one control. This group difference was statistically significant, χ²(1, *N* = 60) = 11.27, *p* < 0.001, and is important to consider when interpreting neurophysiological outcomes.

#### GNG

##### Behavioural outcome (commission errors)

Levene’s test indicated that the assumption of homogeneity of variances was met for GNG errors (Levene’s statistic = 0.02, *p* = 0.898). An independent samples *t*-test revealed that there was no significant main effect of group on GNG errors (*t*(58) = −1.38, *p* = 0.174, *d* = −0.36) between cannabis users (*M* = 5.33 ± 3.22) and controls (*M* = 4.27 ± 2.77).

##### fNIRS

There were no statistically significant differences in oxyHb or deoxyHb average concentrations between cannabis users and controls at any of the channels assessed during Go/No-Go task performance over the entire task epoch and the first 50 seconds of data (see Appendix C for detailed ANOVAsummaries).

##### NASA-TLX

Controls reported significantly higher *temporal demand* (*t*(58) = 2.48, *p* = 0.016, Cohen’s *d* = 0.64, 95% CI: 0.12–1.16) *and frustration* (*t*(58) = 2.29, *p* = 0.025, Cohen’s *d* = 0.59, 95% CI: 0.07–1.11) than cannabis users.

#### Cannabis Stroop task

##### Behavioural outcome (Stoop interference)

Levene’s test indicated that the assumption of homogeneity of variances was met for Stroop Interference (Levene’s statistic = 0.11, *p* = 739). An independent samples *t*-test revealed that there was no significant main effect of group on Stroop Interference (ms; *t*(58) = 0.47, *p* = 0.643, *d* = 0.12) between cannabis users (*M* = 7.47 ± 76.76) and controls (*M* = 16.00 ± 64.41).

##### fNIRS

There were no statistically significant differences in oxyHb or deoxyHb average concentrations between cannabis users and controls at any of the channels assessed during Modified Cannabis Stoop task performance over the entire task epoch and the first 50 seconds of data (see Appendix C for detailed ANOVA summaries).

##### NASA-TLX

Welch’s *t*-test revealed significantly higher *frustration* scores in controls compared to cannabis users, *t*(50.14) = 2.66, *p* = 0.010, Cohen’s *d* = 0.69, 95% CI: 0.16–1.21.

#### SST

##### Behavioural outcome (SSRT)

Levene’s test indicated that the assumption of homogeneity of variances was met for SSRT (Levene’s statistic = 1.49, *p* = 0.228). An independent samples *t*-test revealed that there was no significant main effect of group on SSRT (ms; *t*(58) = −0.735, *p* = 0.465, *d* = −0.19) between cannabis users (*M* = 257.03 ± 62.27) and controls (*M* = 246.83 ± .57).

##### fNIRS

There were no statistically significant differences in oxyHb or deoxyHb average concentrations between cannabis users and controls at any of the channels assessed during SST performance over the entire task epoch and the first 50 seconds of data (see Appendix C for detailed ANOVA summaries).

##### NASA-TLX

No significant group differences were observed on any subscales, all *p*s > 0.05.

#### Exploratory regressions

As an exploratory follow-up, hierarchical linear regressions were conducted within the cannabis user group to examine whether use characteristics predicted behavioural performance and neural activation. No significant associations were found (all overall models *p* > 0.01).

## Discussion

The current study aimed to investigate differences in behavioural and neural responses during cannabis cue-specific inhibitory control tasks between regular cannabis users and non-user controls using fNIRS. Contrary to the hypotheses, the results revealed no significant group differences in behavioural performance or neural activation (i.e. oxyHb and deoxyHb levels) across all three inhibitory reward tasks (i.e. GNG, Cannabis Stroop and SST). Furthermore, there was no significant effect of total lifetime dose, age of onset or current frequency of use on behavioural or neural responses.

The behavioural results are partly in line with previous GNG studies which have largely found no significant difference in errors between cannabis users and controls (Liu et al., 2019). However, deficits in the SST, such as those identified by Liu et al. (2019), may not be present across all cannabis-using populations and might reflect specific patterns of use.

Although fMRI studies have reported neural differences between cannabis users and controls (Hester et al., 2009; Tapert et al., 2007), the fNIRS data in this study did not reveal any group differences in neocortical regions of the PFC, including the OFC. This discrepancy might be due to the inability of fNIRS to access subcortical regions (Pinti et al., 2020), where differential neural activities may have occurred in this sample.

Performance on the Cannabis Stroop Task was similar across groups, contradicting previous research showing poorer performance among heavy or dependent users (Field, 2005). This suggests that the attentional bias defecits reported previously may be specific to more severe patterns of cannabis use, or may reflect effects that are not consistently replicable in non-dependent populations.

The lack of group differences in response to cannabis-related cues is somewhat surprising, given prior findings of increased PFC activation in response to such stimuli (O’Neill et al., 2020; Sehl et al., 2021). However, those studies often used passive cue exposure, whereas the present design embedded cues in active tasks. This suggests that context plays a crucial role in neural cue reactivity. Additionally, it is possible that cue-based reward processes occur at a subcortical level and are thus not detectable using fNIRS (Pinti et al., 2020).

There are several considerations when interpreting these null findings. First, the current sample may not reflect heavy or dependent cannabis use. Participants reported at least weekly cannabis use over the previous 3 months, but this threshold may capture more moderate, non-problematic users. Previous studies that identified significant neural or behavioural effects often included heavier or daily users (Borissova et al., 2022; Field, 2005; Hester et al., 2009; Liu et al., 2019; Sehl et al., 2021; Tapert et al., 2007). Second, the use of a 3-month minimum criterion may have excluded individuals with CUD, reducing the likelihood of detecting neuro-adaptive changes. Prominent addiction models posit that such adaptations emerge with chronic or dependent use (Berridge and Robinson, 2016; Luijten et al., 2014; Zilverstand et al., 2018). This view is supported by evidence that structural and functional brain changes are more likely in dependent populations (Chye et al., 2017, 2019; Lorenzetti et al., 2020, 2024). Stratifying users by diagnostic tools such as the Cannabis Use Disorders Identification Test-Revised (Adamson et al., 2010) may help capture relevant group differences in future studies.

Another limitation is the reliance on self-reported substance use. Although practical and ethically less invasive, self-report can be affected by recall and social desirability bias (Del Boca and Noll, 2000; Hjorthøj et al., 2012; Latkin et al., 2017). No urine screening was conducted to confirm recent cannabis use or rule out other substances. While biological verification offers more accuracy (Huestis et al., 2007; Lorenzetti et al., 2022), such methods remain resource-intensive and are not always feasible in community-based designs.

Methodologically, the SST may have lacked sensitivity. Average stopping accuracy was 18.37%, below the recommended 50%, and the task included fewer stop trials (*n* = 40) than advised (Verbruggen et al., 2019). These factors may have reduced the reliability of SSRT estimation. Task duration may also have influenced fNIRS findings. While the Cannabis Stroop followed block design conventions, the GNG and SST used 4–5-minute blocks. This may have attenuated the haemodynamic response due to filtering effects (Miezin et al., 2000; Scholkmann et al., 2014), despite parameter adjustments. Future research should adopt standard block designs to optimise fNIRS sensitivity.

Future work should stratify cannabis users by severity and usage pattern, broaden the age range and consider multimodal neuroimaging approaches (e.g. fNIRS-fMRI) to assess both cortical and subcortical effects. Longitudinal studies are also needed to clarify developmental trajectories of cannabis-related cognitive and neurophysiological change, as cross-sectional methods may not capture dynamic changes over time (Hanson et al., 2010; Lorenzetti et al., 2024).

In summary, this study found no significant differences in behavioural performance or neural activation between regular cannabis users and non-user controls during cue-specific inhibitory control tasks. These null findings may reflect non-dependent use patterns and task- or method-specific limitations. Further research is needed to examine whether impairments in inhibitory control and brain function emerge specifically with heavier or dependent cannabis use.

## Supplemental Material

sj-docx-1-jop-10.1177_02698811251358814 – Supplemental material for No differences in neural responses or performance during cannabis cue-specific inhibitory control tasks between recreational cannabis users and non-users: Insights from fNIRS Supplemental material, sj-docx-1-jop-10.1177_02698811251358814 for No differences in neural responses or performance during cannabis cue-specific inhibitory control tasks between recreational cannabis users and non-users: Insights from fNIRS by Christopher R. Pickering, Valentina Lorenzetti, Andrew Jones, Martin Guest, Paul Christiansen and Carl A. Roberts in Journal of Psychopharmacology
