# Supplementary material for: No differences in neural responses or performance during cannabis cue-specific inhibitory control tasks between recreational cannabis users and non-users: Insights from fNIRS
Source: J Psychopharmacol. 2025 Oct 2;40(3):422–8. doi: 10.1177/02698811251358814 (PMC13242547; doi:10.1177/02698811251358814)
Supplement: sj-docx-1-jop-10.1177_02698811251358814 – Supplemental material for No differences in neural responses or performance during cannabis cue-specific inhibitory control tasks between recreational cannabis users and non-users: Insights from fNIRS [file sj-docx-1-jop-10.1177_02698811251358814.docx]

**Appendix A - Expanded Methods and Data Processing**

**Questionnaires**

**Background Drug Use Questionnaire (Montgomery et al., 2005)**

A background drug use questionnaire was used to examine current and historic patterns of drug use amongst participants. The questionnaire assesses numerous indices of the use of cannabis and other drugs, along with the respondents' age. A method similar to the one employed by Montgomery et al. (2005) was utilised to estimate the total lifetime consumption of cannabis and other substances.

Cannabis amounts were quantified in Standard Joint Units (SJUs), where 1 SJU equates to 0.25 grams of cannabis containing approximately 7 mg of Δ9-THC, as outlined by Kögel et al. (2017). For powder substances, dosages were measured in grams, with 1 line corresponding to 0.2 grams, following methods described in Liu et al. (2021). Ecstasy pills were estimated as containing 150mg of MDMA per pill, based on averages stated in the European Drug Report (European Monitoring Centre for Drugs and Drug Addiction [EMCDDA], 2021).

**Mood adjective checklist (MAC) (Fisk & Warr, 1996)**

State anxiety, arousal, and hedonic tone were measured using the state mood adjective checklist developed by Fisk and Warr (1996). Participants rated their feelings at the time of testing on a 5‐point Likert scale from 1 (not at all) to 5 (extremely) on several items related to each subscale. A high score on each subscale indicates increased anxiety, arousal, and hedonic tone.

**North American Space Agency-Task Load Index (NASA-TLX, Hart & Staveland, 1988)**

This is a multi-dimensional scale, consisting of six sub-scales (i.e., mental demand, physical demand, temporal demand, performance rating, effort and frustration). After each behavioural task, participants were required to place a mark on a line indicating where they perceive their demand to be on the scale from 0-100 in increments of 5. As this study did not involve physical exertion, physical demand was not included in analyses.

**Raven’s Progressive Matrices (RPM) (Penrose & Raven, 1936)**

Participants completed sets D & E (12 items in each) of Raven’s Progressive Matrices (RPM; Penrose & Raven, 1936). RPM is a non-verbal measure of fluid intelligence in which participants are required to select - from 8 options - the missing element from a pattern (presented in 3x3 matrices). Higher scores indicate higher levels of fluid intelligence.

**AUDIT-C (Bush et al., 1998)**

AUDIT-C is the standard 3-item version of the Alcohol Use Disorders Identification Test [AUDIT]. It measures frequency of consumption, typical units on a drinking occasion, and frequency of binge drinking (Bush et al., 1998). Summation of the responses to these three items results in a total score ranging from 0-12. A total of 5 or more is indicative of hazardous drinking. Cronbach’s Alpha for the scale in this sample was *α*=.83, indicating good internal consistency

**Behavioural Tasks**

**Go/No-Go Task (Logan & Cowan, 1984)**

This task measured action restraint, a subcomponent of inhibitory control. The task used in this experiment was based on the food-specific Go/No-Go task developed by Houben and Jansen et al. (2011), but with food cues replaced by images from a cannabis cue stimulus set developed by McCattee et al. (2021).

On each trial, participants were presented with an image in either portrait or landscape orientation. Their task was to press the space bar when the image was a landscape (Go) and inhibit their response if the image was portrait (No-Go). Each trial began with the presentation of a fixation cross ('+') for 500 ms. Following this, one of the two stimuli - portrait-orientated pictures or landscape-orientated pictures - appeared in the centre of the screen. 75% of trials were 'Go' trials in which the stimulus remained on screen until participants provided a response or until a 2000ms timeout had elapsed.

The task was split into three blocks:

Firstly, a practice block of 10 trials intended to familiarise participants with the task. This was followed by an additional practice block of 30 trials with landscape images appearing on every trial, intending to produce pre-potent/dominant responses which participants were required to inhibit. Data from these initial practice blocks were not recorded. The remaining block consisted of 160 trials: 120 'Go' trials and 40 'No-Go' trials presented in a random order. The completion of the task took approximately 5 minutes. The primary outcome measure for the analysis of performance was No-Go errors.

**Supplementary Figure A1**

*Schematic Diagram of the Go/No-Go task*


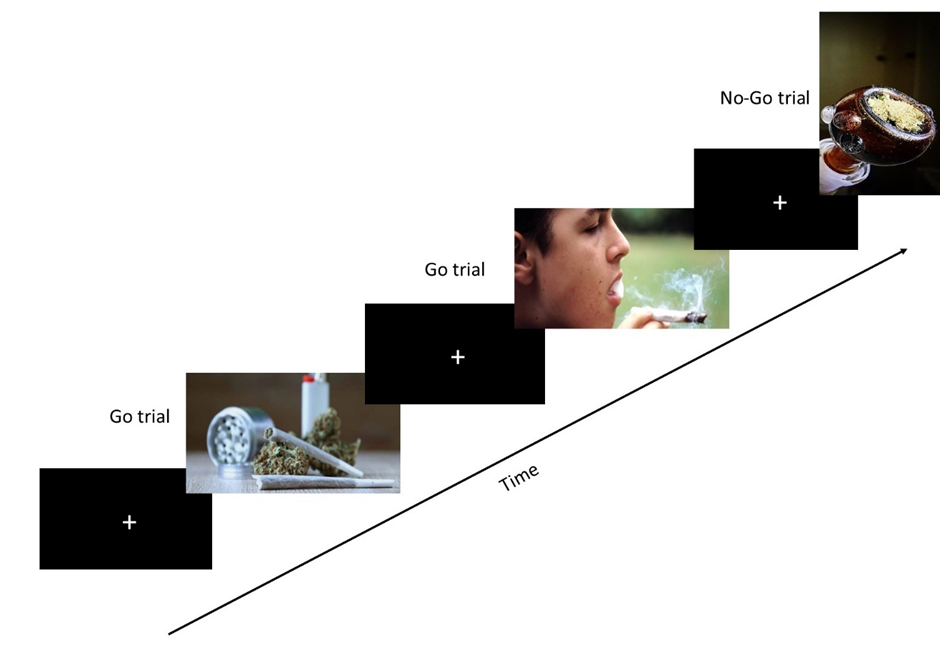


**Cannabis Stroop Task (Field, 2005)**

The Cannabis Stroop is a variation of the Emotive Stroop (Williams et al., 1996) used to measure attentional bias in which interference - indicated by slower reaction time - is a consequence of the emotional content of the words. In this task, participants saw words (cannabis words, or neutral words) presented in one of four colours. Their task was to indicate the colour the word was presented in, while ignoring the words themselves – by pressing a corresponding key on the keyboard. These individual keys had a sticker in their corresponding colour: A for Red words, S for Blue words, K for Yellow words, and L for Green words.

Cannabis-related and matched neutral words were used in the modified Stroop task. The word sets were based on those used in a previous study (Field, 2005). Twenty cannabis-related words were matched for length and syllables with words that described a characteristic of the natural environment (e.g., cannabis–seawater, resin-holly, ganja-inlet).

The task was split into five blocks:

Firstly, a practice block of 10 trials intended to familiarise participants with the task. Data from this practice block were not recorded. Words used in this task were not related to cannabis or the natural environment. This was followed by four alternating blocks consisting of either 20 cannabis-related word trials or 20 neutral word trials. These were counterbalanced across participants. Completion of the task took approximately 5 minutes.

The primary outcome for analysis was Stroop Interference Reaction Time (i.e., the time taken to correctly identify the colours of cannabis words minus the time taken to correctly identify the colours of neutral words, excluding errors and anticipatory responses below 150ms).

**Supplementary Figure A2**

*Flow Chart of the Modified Cannabis Stroop task*


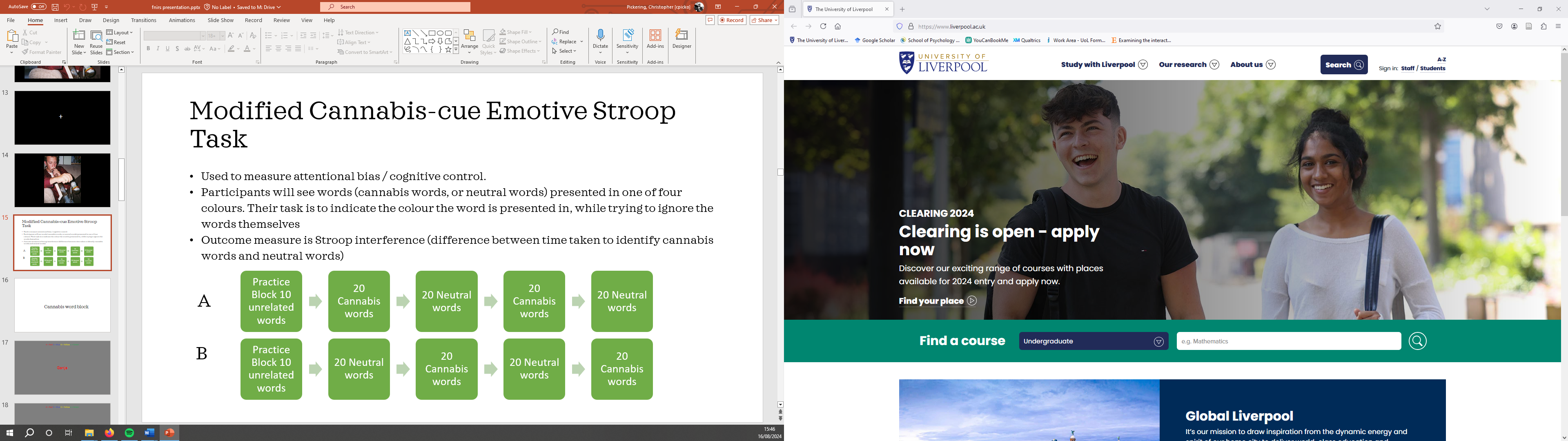


Note. Streams A and B represent counterbalancing across participants

**Supplementary Figure A3**

*Example Trials from the Modified Cannabis Stroop task*


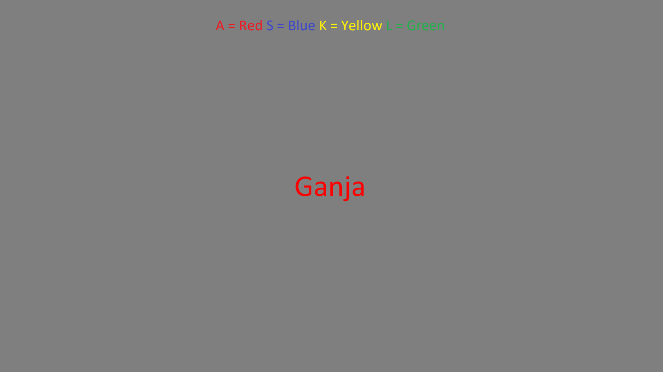

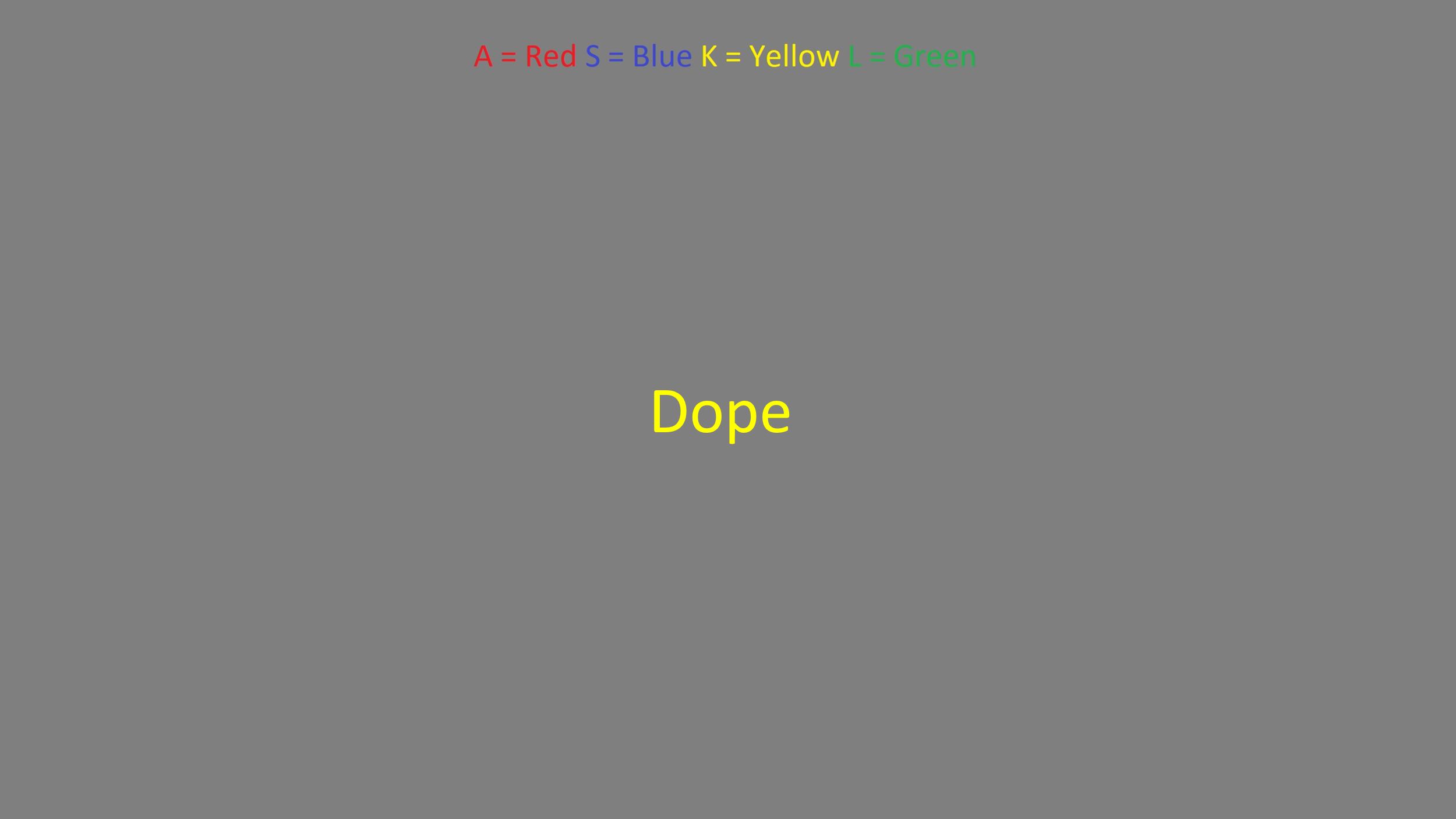


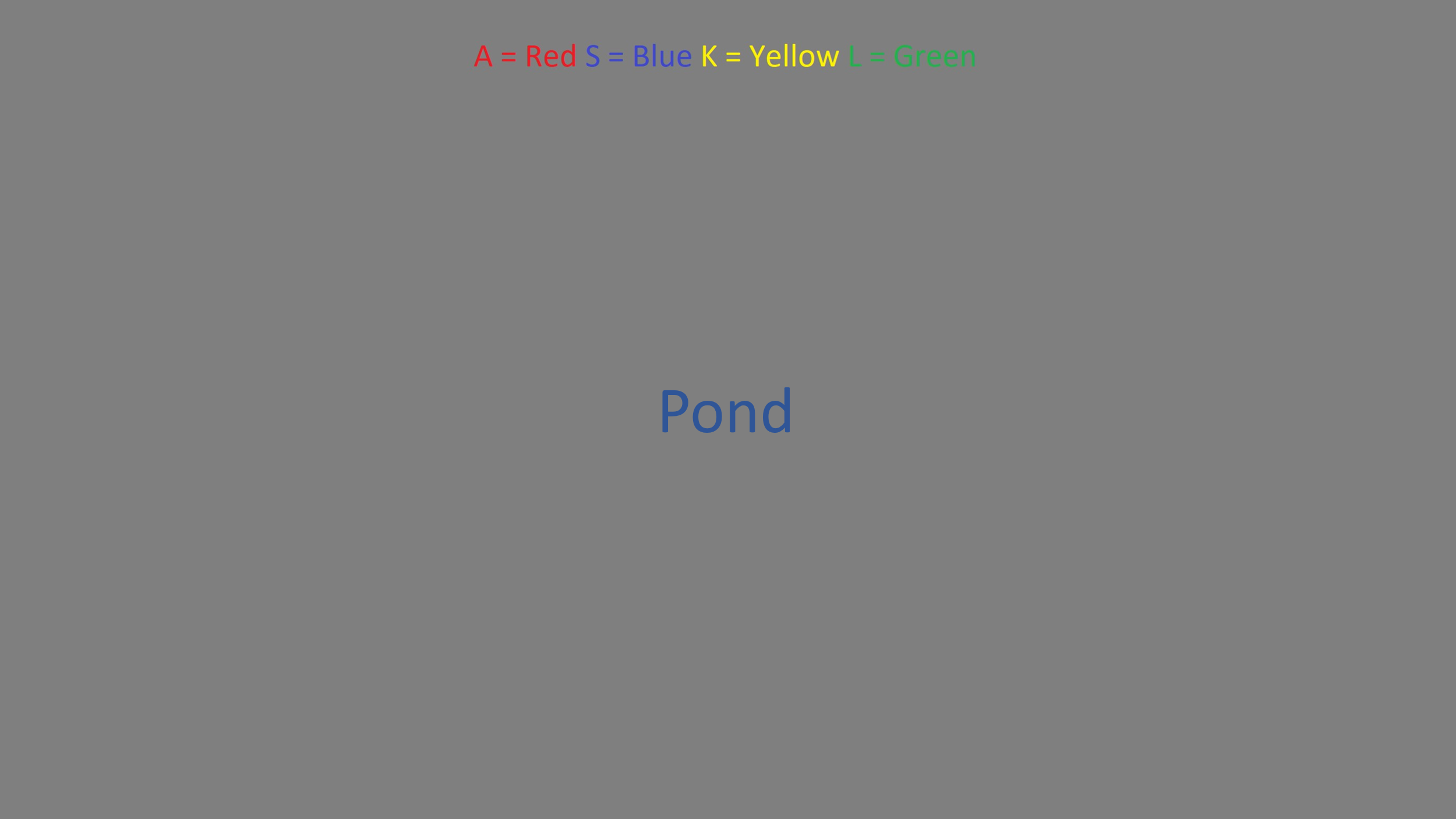

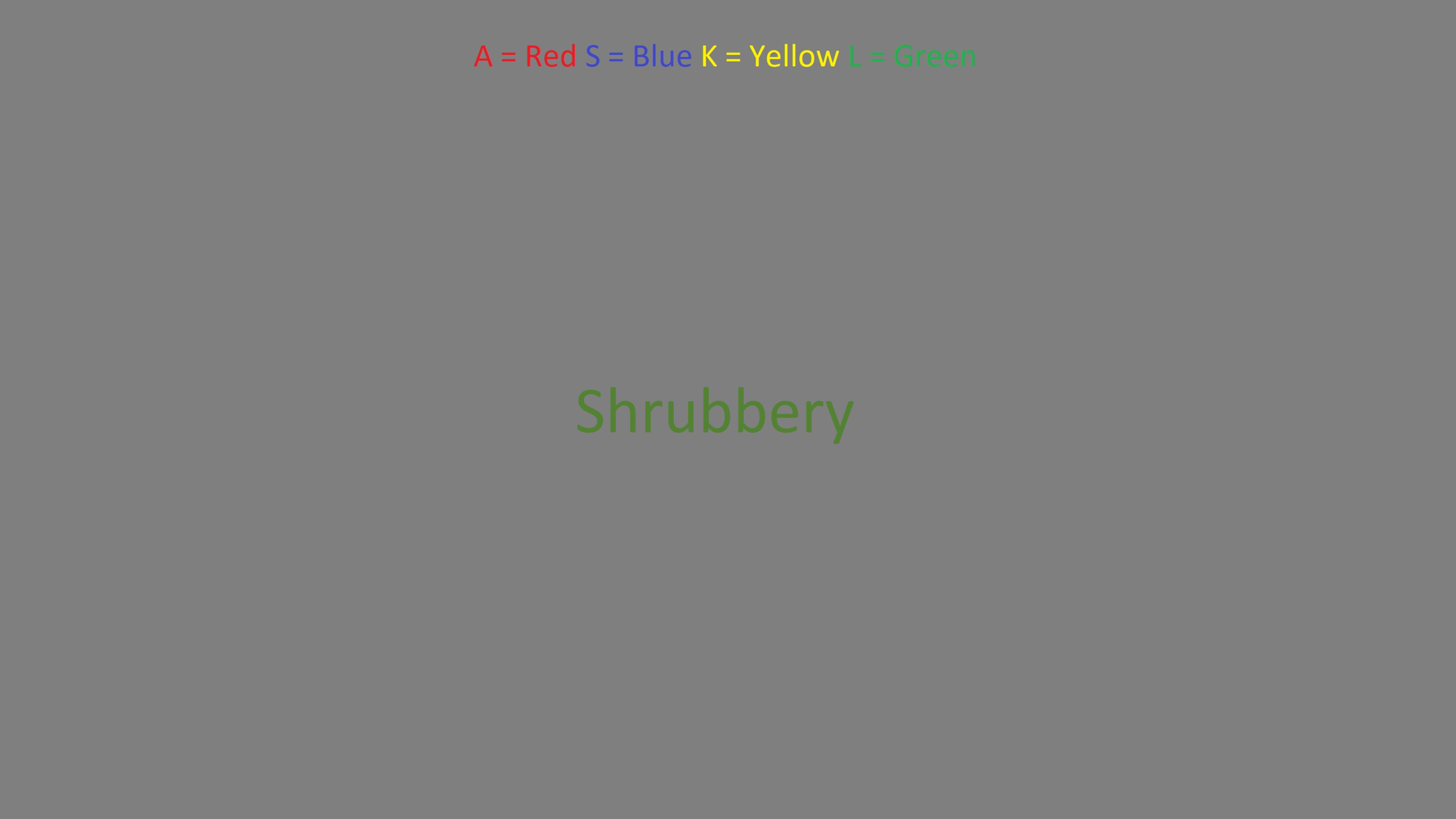


Note. First row is ‘Cannabis’ words. The second row is ‘neutral’ words

**Stroop Task Word Lists**

The following are the word lists used in the Stroop task, divided by condition.

**Practice Words**

1. Hello
2. Football
3. Cloud
4. Book
5. Training
6. Aeroplane
7. Building
8. Box
9. Funnel
10. Tiger
11. Typewriter
12. Rainfall
13. Television
14. Headphones
15. Running
16. Recording
17. Sorting
18. Road map
19. Bottle
20. Fixture

**Cannabis-Related Words**

1. Cannabis
2. Dealer
3. Dope
4. Ganja
5. Grass
6. Hash
7. Joint
8. Marijuana
9. High
10. Pot
11. Resin
12. Rizla
13. Roach
14. Spliff
15. Stoned
16. Weed
17. Bong
18. Pipe
19. Blunt
20. Skunk

**Neutral Words**

1. Seawater
2. Ravine
3. Cove
4. Inlet
5. Winds
6. Sand
7. Cliff
8. Shrubbery
9. Lake
10. Fog
11. Holly
12. Daisy
13. Swamp
14. Trench
15. Cactus
16. Hill
17. Pond
18. Rain
19. Earth
20. Trees

**Stop-Signal Task (Verbruggen & Logan, 2008)**

This task measured action cancellation, the ability to inhibit a response already in progress . The task used in the experiment was based on the protocol developed by Verbruggen et al. (2008) with images from a cannabis cue stimulus set developed by McCattee et al. (2021). On each trial, participants were presented with an image in either portrait or landscape orientation. Their task was to press the 'D' key when the image was portrait and to press the 'K' key when the image was landscape. On certain trials, an '=' sign overlaid the image just after it was presented. This was a stop signal. If participants saw an '=' sign, they were required to inhibit their response on that trial. Each trial began with the presentation of a fixation cross (‘+’) for 500 ms.

Following this, one of two types of stimuli (i.e., either portrait-orientated pictures or landscape-orientated pictures) appeared in the centre of the screen. 120 of the trials were ‘go’ trials in which the stimulus remained on the screen until participants provided a response or until a 1500ms timeout had elapsed. The remaining 40 trials (presented at random) were stop-signal trials. On these trials, a stop signal indicated by a red ‘=’ sign, appeared over the stimuli, requiring participants to inhibit their response.

The stop signal delay (SSD) was dynamically adjusted using a 3-down, 1-up staircase procedure to balance task difficulty based on participant performance. At the start of the task, the SSD was initialised to 250 ms. Adjustments to the SSD were made as follows:

- Successful inhibition: If the participant successfully inhibited their response to a stop signal, the SSD increased, making inhibition more challenging in subsequent trials.
- Failed inhibition: If the participant failed to inhibit their response, the SSD decreased, making inhibition easier in subsequent trials.

The staircase procedure followed a 3-down, 1-up rule, such that the SSD increased after three consecutive successful inhibitions and decreased after a single failed inhibition. This approach ensured that the SSD dynamically adapted to stabilise performance at a consistent level across trials.

Step sizes of 1, 0.75, 0.5, and 0.25 (in ms) were used, with larger adjustments early in the task and finer adjustments as the staircase progressed. The range of SSDs was restricted between a minimum of 50 ms and a maximum of 1150 ms to prevent extreme values.

The task was split into three blocks:

Firstly, a practice block of 10 trials intended to familiarise participants with the task. This was followed by an additional practice block of 30 trials with no stop-signals, intending to produce pre-potent/dominant response which participants were required to inhibit. Data from these initial practice blocks were not recorded. The remaining experimental block consisted of 160 trials: 120 ‘Go’ trials and 40 ‘Stop-signal’ trials presented in a random order.

Completion of the task took approximately 5 minutes. The primary outcome measure for analysis of performance was the Stop Signal Reaction Time (SSRT), measured in ms, calculated using the integration method described by Verbruggen et al. (2019). SSRT is the unobserved latency of inhibition, with longer SSRTs indicative of poorer inhibitory control.

**Supplementary Figure A4**

*Schematic Diagram of the Go/No-Go task*


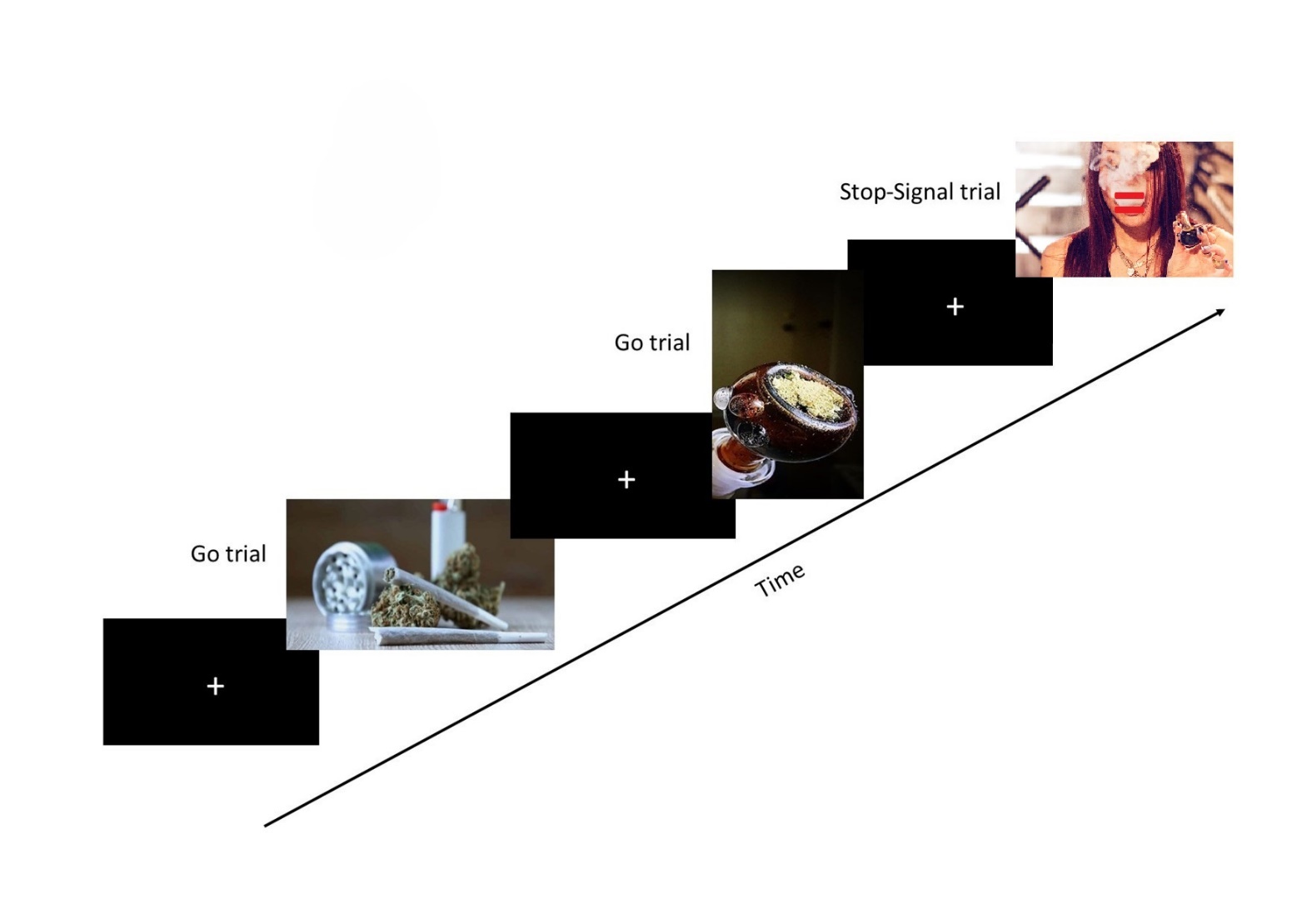


**fNIRS Data Collection**

For fNIRS data collection participants wore a flexible, anatomically registered EASYCAP head-cap (EASYCAP GmbH, Germany), to which were attached 8 light-emitting source fibres, and 6 light-detecting fibres (Gowerlabs Ltd, London, UK). this custom array of sources and detectors constitutes a total of 18 channels and one short-separation channel, which was excluded from use. As can be seen in Figure A5, this array was positioned to examine haemodynamic activity in several key regions of the prefrontal cortex, including the dorsolateral prefrontal cortex (DLPFC), ventromedial prefrontal cortex (vmPFC), medial prefrontal cortex (mPFC), orbitofrontal cortex (OFC), anterior prefrontal cortex (aPFC), and inferior frontal gyrus (IFG). These areas are implicated in cognitive functions such as inhibitory control, decision-making, and reward signalling (Howard & Kahnt, 2021; Menon & D’Esposito, 2022). Data was acquired with the NTS2 fNIRS system (Gowerlabs Ltd. London, UK) which uses two continuous wavelengths of light at 780 nm and 850 nm for assessing oxyHb and deoxyHb, and has a sampling rate of 10 Hz.

**fNIRS Data Analysis: Pre-Processing**

Initial fNIRS analyses were performed using Homer3, an open-source software (Huppert et al., 2009), and implemented in MATLAB (The Mathworks Inc., 2022). The NIRS signal was initially transformed into optical density. Subsequently, motion artifacts in the signal were identified and corrected using a hybrid approach combining spline interpolation and wavelet filtering (De Lorenzo et al., 2019). Automatic channel pruning using a signal to noise ratio threshold of 12.5 was used. Then a high-pass filter with variable cutoff frequencies (0.001Hz for the Go/No-Go and Stop Signal Task and 0.01Hz for the Stroop, based on length of data) was used to eliminate baseline drift and low-frequency oscillations, as well as a 0.45Hz low-pass filter to mitigate the influence of heartbeat pulsations and high-frequency noise. The modified Beer-Lambert law, employing a differential pathlength factor of 6.0, 5.0 (calculated as per Scholkmann & Wolf, 2013), was applied to compute changes in haemoglobin concentrations. Visual inspection was performed to confirm the effectiveness of these filters. Participant data were excluded from individual task analyses if fewer than 60% of channels (i.e., fewer than 11) were usable. This criterion resulted in the exclusion of one participant from the Cannabis Stroop task and two participants from the Go/No-Go task.

To derive an average response to the tasks across each participant, the hemodynamic response function (HRF) was measured using a block average for the task epoch. The output from Homer3 was then exported to SPSS for further analyses. A supplementary analysis of the first 50 seconds of the Go/No-Go and Stop Signal Task (using a high-pass filter of 0.01HZ) was conducted to control for flattening and stabilisation of fNIRS signals by filtering over the 4-5-minute duration of these tasks.

**Supplementary Figure A5**

*fNIRS Array Sensitivity Map with covering regions of the prefrontal cortex, and orbitofrontal cortex*


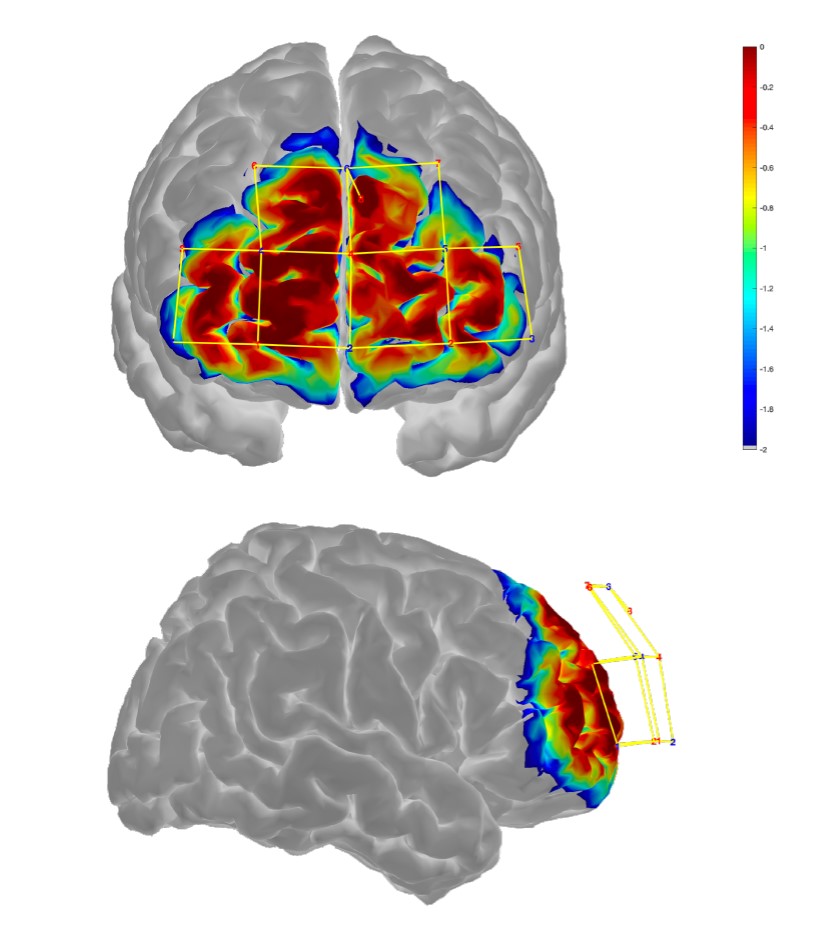


**Supplementary Figure A6**

*fNIRS channel labels*

**
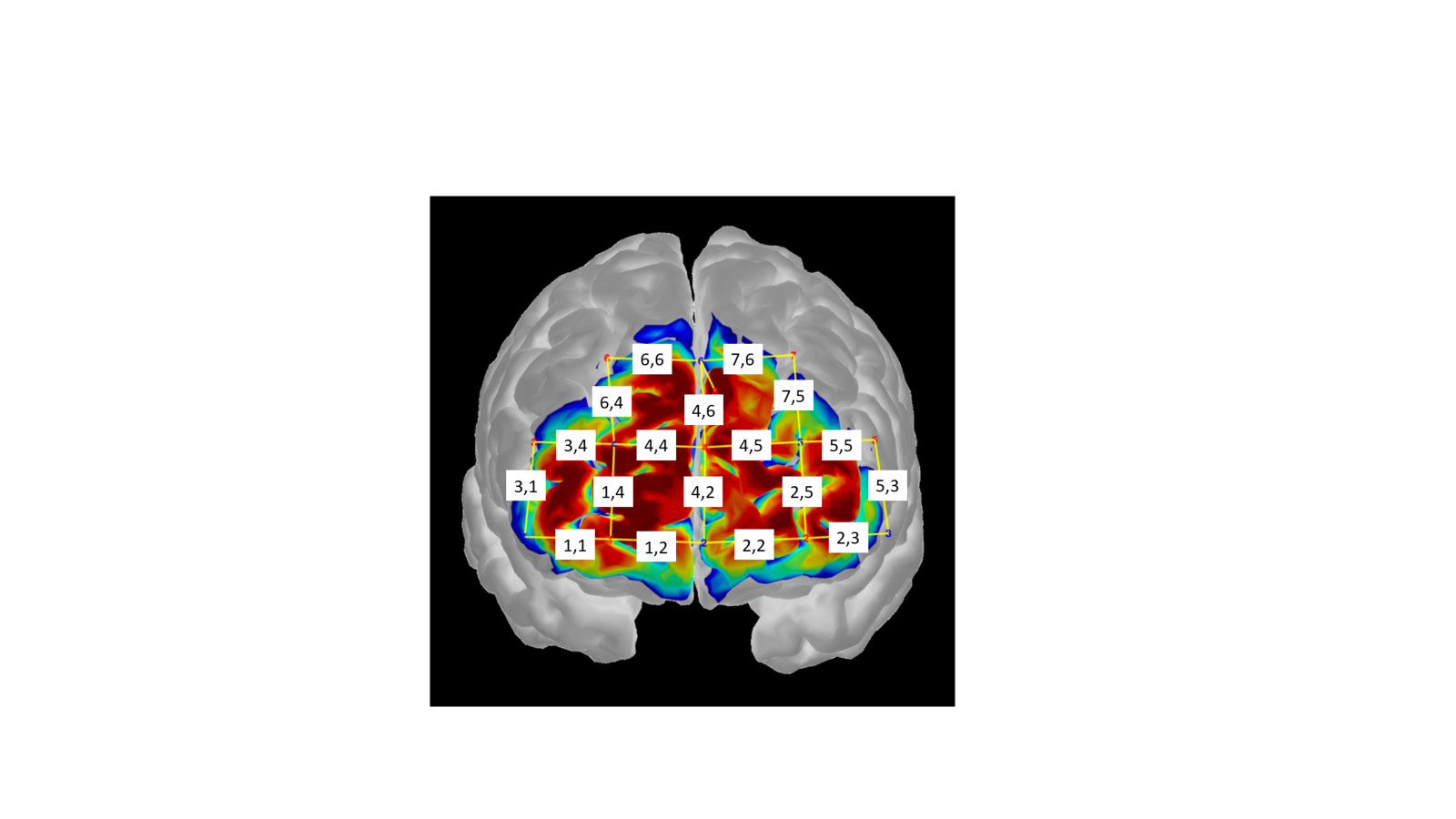
**

Note. Channels are labelled in the format ‘source, detector’

Source optodes are in red, detectors are in blue

**Appendix B** – **Substance Use Tables**

**Supplementary Table B1**

*Descriptive Statistics for Abstinence and Dose on Last Use by Substance and Group*

| Substance | Group | N | Mean Abstinence (Days) | SD | Range | Mean Dose on Last Use | SD | Unit |
| --- | --- | --- | --- | --- | --- | --- | --- | --- |
| Alcohol | Control | 18 | 3.33 | 2.09 | 1–8 | 8.78 | 6.88 | UK units |
| Alcohol | Cannabis | 18 | 3.50 | 2.38 | 1–9 | 9.76 | 7.76 | UK units |
| Cannabis | Cannabis | 26 | 2.08 | 1.52 | 1–7 | 2.51 | 2.48 | SJUs |
| Cocaine | Cannabis | 3 | 6.00 | 4.58 | 1–10 | 0.225 | 0.115 | grams |
| MDMA | Cannabis | 4 | 3.25 | 2.22 | 1–6 | 363.75 | 164.08 | mg |
| Ketamine | Cannabis | 7 | 5.71 | 4.23 | 1–10 | 0.53 | 0.41 | grams |

Note. Valid Ns vary by substance. No control participants reported cannabis or illicit substance use.

**Supplementary Table B2**

*Self-Reported Substance Use Quantity in the Past 30 Days by Group*

| Substance | Group | N | Mean Use (Last 30 Days) | SD | Range | Unit |
| --- | --- | --- | --- | --- | --- | --- |
| Cannabis | Cannabis | 29 | 42.76 | 43.08 | 2–180 | SJUs |
| Cocaine | Cannabis | 8 | 0.95 | 0.62 | 0.2–2.0 | Grams |
| MDMA | Cannabis | 5 | 651.00 | 657.84 | 130–1800 | Milligrams |
| Ketamine | Cannabis | 12 | 1.89 | 2.65 | 0.125–9.6 | Grams |
| LSD | Cannabis | 1 | 660.00 | — | — | Micrograms |
| Cocaine | Control | 1 | 0.20 | — | — | Grams |

Note. Control participants reported no cannabis or other illicit substance use in the past 30 days, with the exception of one individual reporting minimal cocaine use. Values reflect self-reported quantity consumed over the 30 days preceding testing. Units were standardised per substance.

**Supplementary Table B3**

*Self-reported frequency of substance use within the past 3 months, by Group*

| Substance | Group | Never Used | Used Previously* | <Monthly | At Least Monthly | At Least Weekly | Daily | Total N |
| --- | --- | --- | --- | --- | --- | --- | --- | --- |
| Alcohol | Control | 4 | – | 3 | 12 | 11 | 0 | 30 |
|  | Cannabis | 0 | – | 2 | 11 | 17 | 0 | 30 |
| Cannabis | Control | 23 | 7 | 0 | 0 | 0 | 0 | 30 |
|  | Cannabis | 0 | – | 0 | 6 | 12 | 10 | 30 |
| Amphetamine | Control | 30 | – | 0 | 0 | 0 | 0 | 30 |
|  | Cannabis | 26 | 3 | 0 | 1 | 0 | 0 | 30 |
| Cocaine | Control | 25 | 3 | 2 | 0 | 0 | 0 | 30 |
|  | Cannabis | 11 | 6 | 6 | 6 | 1 | 0 | 30 |
| Ecstasy (MDMA) | Control | 27 | 3 | 0 | 0 | 0 | 0 | 30 |
|  | Cannabis | 8 | 14 | 7 | 1 | 0 | 0 | 30 |
| Ketamine | Control | 26 | 2 | 1 | 1 | 0 | 0 | 30 |
|  | Cannabis | 6 | 6 | 7 | 9 | 2 | 0 | 30 |
| LSD | Control | 29 | 1 | 0 | 0 | 0 | 0 | 30 |
|  | Cannabis | 13 | 15 | 1 | 1 | 0 | 0 | 30 |
| Mushrooms | Control | 29 | 1 | 0 | 0 | 0 | 0 | 30 |
|  | Cannabis | 16 | 11 | 3 | 0 | 0 | 0 | 30 |
| Poppers | Control | 28 | 1 | 1 | 0 | 0 | 0 | 30 |
|  | Cannabis | 15 | 7 | 7 | 1 | 0 | 0 | 30 |
| DMT | Control | 30 | 0 | 0 | 0 | 0 | 0 | 30 |
|  | Cannabis | 26 | 4 | 0 | 0 | 0 | 0 | 30 |
| Mephedrone | Control | 29 | 1 | 0 | 0 | 0 | 0 | 30 |
|  | Cannabis | 27 | 3 | 0 | 0 | 0 | 0 | 30 |
| Other | Control | 30 | 0 | 0 | 0 | 0 | 0 | 30 |
|  | Cannabis | 24 | 5 | 1 | 0 | 0 | 0 | 30 |

* “Used Previously” = used at some point in lifetime but not in the past 3 months.

**Appendix C – ANOVA Summaries and Behavioral Regressions**

**Supplementary Table C1**

*fNIRS ANOVA Results for Go/No-Go Task (Full Task Epoch)*

| **Channel** | **SS** | **df** | **MS** | **F** | **p** | **ηp2** | **Assumption of Homogeneity Met** |
| --- | --- | --- | --- | --- | --- | --- | --- |
| **OxyHb** |  |  |  |  |  |  |  |
| Channel 1,1 | .091 | 1,53 | .091 | .051 | .823 | .001 | Yes |
| Channel 1,2 | 1.360 | 1,55 | 1.360 | .71 | .403 | .013 | Yes |
| Channel 1,4 | .589 | 1,51 | .589 | .38 | .539 | .007 | Yes |
| Channel 2,2 | .194 | 1,54 | .194 | .08 | .776 | .002 | Yes |
| Channel 2,3 | 3.310 | 1,53 | 3.310 | 1.81 | .185 | .033 | Yes |
| Channel 2,5 | .236 | 1,52 | .236 | .14 | .708 | .003 | Yes |
| Channel 3,1 | .369 | 1,51 | .369 | .42 | .521 | .008 | Yes |
| Channel 3,4 | .272 | 1,53 | .272 | .26 | .602 | .005 | Yes |
| Channel 4,2 | 6.001 | 1,49 | 6.001 | 4.92 | .031 | .091 | Yes |
| Channel 4,4 | 2.912 | 1,52 | 2.912 | 2.16 | .148 | .040 | Yes |
| Channel 4,5 | 4.089 | 1,54 | 4.089 | 3.48 | .068 | .061 | Yes |
| Channel 4,6 | .288 | 1,47 | .288 | .09 | .765 | .002 | Yes |
| Channel 5,3 | .786 | 1,51 | .786 | .57 | .455 | .011 | Yes |
| Channel 5,5 | .545 | 1,38.41 | .545 | .51 | .478 | .010 | No* |
| Channel 6,4 | .105 | 1,50 | .105 | .09 | .772 | .002 | Yes |
| Channel 6,6 | 3.871 | 1,41 | 3.871 | 2.09 | .156 | .049 | Yes |
| Channel 7,5 | .017 | 1,52 | .017 | .01 | .914 | .000 | Yes |
| Channel 7,6 | .355 | 1,45 | .355 | .12 | .728 | .003 | Yes |
| **DeOxyHB** |  |  |  |  |  |  |  |
| Channel 1,1 | .314 | 1,53 | .314 | .76 | .386 | .014 | Yes |
| Channel 1,2 | .025 | 1,55 | .025 | .09 | .772 | .002 | Yes |
| Channel 1,4 | .087 | 1,51 | .087 | .27 | .605 | .005 | Yes |
| Channel 2,2 | .118 | 1,54 | .118 | .18 | .673 | .003 | Yes |
| Channel 2,3 | .355 | 1,53 | .355 | .62 | .434 | .012 | Yes |
| Channel 2,5 | .100 | 1,52 | .100 | .28 | .601 | .005 | Yes |
| Channel 3,1 | .001 | 1,51 | .001 | .00 | .953 | .000 | Yes |
| Channel 3,4 | .011 | 1,53 | .011 | .04 | .848 | .001 | Yes |
| Channel 4,2 | 1.647 | 1,41.07 | 1.647 | 6.17 | .017 | .106 | No |
| Channel 4,4 | 3.359 | 1,53 | 3.359 | 3.99 | .051 | .070 | Yes |
| Channel 4,5 | 1.136 | 1,54 | 1.136 | 1.84 | .180 | .033 | Yes |
| Channel 4,6 | .113 | 1,47 | .113 | .061 | .807 | .001 | Yes |
| Channel 5,3 | .864 | 1,51 | .864 | 1.52 | .223 | .029 | Yes |
| Channel 5,5 | .624 | 1,50 | .624 | 2.56 | .116 | .049 | No* |
| Channel 6,4 | .097 | 1,50 | .097 | .17 | .687 | .003 | Yes |
| Channel 6,6 | 1.148 | 1,41 | 1.148 | .78 | .381 | .019 | Yes |
| Channel 7,5 | .174 | 1,52 | .174 | .37 | .544 | .007 | Yes |
| Channel 7,6 | .007 | 1,45 | .007 | .00 | .949 | .000 | Yes |

**Note:**

- SS = Sum of Squares
- df = Degrees of Freedom
- MS = Mean Square
- F = F-ratio
- p = p-value
- *Channels with a "No" under "Assumption of Homogeneity Met" had their results verified using Welch's F-statistic.

**Supplementary Table C2**

*fNIRS ANOVA Results for Modified Cannabis Stroop Task (Full Task Epoch)*

| **Channel** | **SS** | **df** | **MS** | **F** | **p** | **ηp2** | **Assumption of Homogeneity Met** |
| --- | --- | --- | --- | --- | --- | --- | --- |
| **OxyHb** |  |  |  |  |  |  |  |
| Channel 1,1 | 1.434 | 1,57 | 1.434 | 1.43 | .237 | .024 | Yes |
| Channel 1,2 | .038 | 1,57 | .038 | .04 | .849 | .001 | Yes |
| Channel 1,4 | .049 | 1,56 | .049 | .10 | .755 | .002 | Yes |
| Channel 2,2 | 1.532 | 1,56 | 1.532 | 1.39 | .244 | .024 | Yes |
| Channel 2,3 | .114 | 1,57 | .114 | .20 | .661 | .003 | Yes |
| Channel 2,5 | .054 | 1,57 | .054 | .10 | .749 | .002 | Yes |
| Channel 3,1 | .346 | 1,56 | .346 | .39 | .536 | .007 | Yes |
| Channel 3,4 | .140 | 1,57 | .140 | .17 | .682 | .003 | Yes |
| Channel 4,2 | .028 | 1,56 | .028 | .04 | .852 | .001 | Yes |
| Channel 4,4 | .199 | 1,56 | .199 | .27 | .606 | .005 | Yes |
| Channel 4,5 | .023 | 1,55 | .023 | .02 | .878 | .000 | Yes |
| Channel 4,6 | 2.384 | 1,53 | 2.384 | 1.17 | .284 | .022 | Yes |
| Channel 5,3 | .572 | 1,55 | .572 | .33 | .568 | .006 | Yes |
| Channel 5,5 | .712 | 1,55 | .712 | 1.25 | .269 | .022 | Yes |
| Channel 6,4 | 1.478 | 1,57 | 1.478 | 1.15 | .288 | .020 | Yes |
| Channel 6,6 | 1.731 | 1,48 | 1.731 | .27 | .609 | .006 | Yes |
| Channel 7,5 | 2.833 | 1,54 | 2.833 | 1.33 | .253 | .024 | Yes |
| Channel 7,6 | .992 | 1,46 | .992 | .65 | .425 | .014 | Yes |
| **DeOxyHB** |  |  |  |  |  |  |  |
| Channel 1,1 | .584 | 1,57 | .584 | 2.84 | .097 | .047 | Yes |
| Channel 1,2 | .001 | 1,57 | .001 | .00 | .953 | .000 | Yes |
| Channel 1,4 | .103 | 1,56 | .103 | 1.412 | .239 | .025 | Yes |
| Channel 2,2 | .061 | 1,56 | .061 | .40 | .528 | .007 | Yes |
| Channel 2,3 | .068 | 1,57 | .068 | .54 | .464 | .009 | Yes |
| Channel 2,5 | .046 | 1,57 | .046 | .37 | .547 | .006 | Yes |
| Channel 3,1 | .208 | 1,56 | .208 | 1.11 | .298 | .019 | Yes |
| Channel 3,4 | .077 | 1,57 | .077 | .50 | .482 | .009 | Yes |
| Channel 4,2 | .107 | 1,56 | .107 | .86 | .359 | .015 | Yes |
| Channel 4,4 | .006 | 1,56 | .006 | .03 | .859 | .001 | Yes |
| Channel 4,5 | .077 | 1,55 | .077 | .33 | .569 | .006 | Yes |
| Channel 4,6 | .093 | 1,53 | .093 | .19 | .666 | .004 | Yes |
| Channel 5,3 | .434 | 1,55 | .434 | .62 | .435 | .011 | Yes |
| Channel 5,5 | .035 | 1,43.12 | .035 | .26 | .615 | .005 | No* |
| Channel 6,4 | 1.281 | 1,57 | 1.281 | 1.81 | .184 | .031 | Yes |
| Channel 6,6 | .040 | 1,37.58 | .040 | .051 | .822 | .001 | No* |
| Channel 7,5 | 1.099 | 1,54 | 1.099 | 1.46 | .233 | .026 | Yes |
| Channel 7,6 | .153 | 1,46 | .153 | .52 | .476 | .011 | Yes |

**Note:**

- SS = Sum of Squares
- df = Degrees of Freedom
- MS = Mean Square
- F = F-ratio
- p = p-value
- *Channels with a "No" under "Assumption of Homogeneity Met" had their results verified using Welch's F-statistic.

**Supplementary Table C3**

*fNIRS ANOVA Results for Stop-Signal Task (Full Task Epoch)*

| **Channel** | **SS** | **df** | | **MS** | **F** | **p** | **ηp2** | **Assumption of Homogeneity Met** |
| --- | --- | --- | --- | --- | --- | --- | --- | --- |
| **OxyHb** |  |  |  | |  |  |  |  |
| Channel 1,1 | 1.123 | 1,57 | | 1.123 | .58 | .451 | .010 | Yes |
| Channel 1,2 | .052 | 1,57 | | .052 | .03 | .854 | .001 | Yes |
| Channel 1,4 | .010 | 1,55 | | .010 | .01 | .918 | .000 | Yes |
| Channel 2,2 | 3.780 | 1,54 | | 3.780 | 2.19 | .145 | .039 | Yes |
| Channel 2,3 | .001 | 1,56 | | .001 | .00 | .976 | .000 | Yes |
| Channel 2,5 | .744 | 1,56 | | .744 | .65 | .425 | .011 | Yes |
| Channel 3,1 | .767 | 1,56 | | .767 | .54 | .467 | .009 | Yes |
| Channel 3,4 | .010 | 1,57 | | .010 | .01 | .917 | .000 | Yes |
| Channel 4,2 | .563 | 1,57 | | .563 | .55 | .462 | .010 | Yes |
| Channel 4,4 | 2.820 | 1,55 | | 2.820 | 2.22 | .142 | .039 | Yes |
| Channel 4,5 | .825 | 1,57 | | .825 | .60 | .444 | .010 | Yes |
| Channel 4,6 | 6.172 | 1,51 | | 6.172 | 5.13 | .028 | .091 | Yes |
| Channel 5,3 | .000 | 1,57 | | .000 | .00 | .990 | .000 | Yes |
| Channel 5,5 | .546 | 1,56 | | .546 | .67 | .417 | .012 | Yes |
| Channel 6,4 | .422 | 1,58 | | .422 | .38 | .540 | .007 | Yes |
| Channel 6,6 | .118 | 1,46 | | .118 | .06 | .807 | .001 | Yes |
| Channel 7,5 | .004 | 1,55 | | .004 | .00 | .947 | .000 | Yes |
| Channel 7,6 | .284 | 1,39.32 | | .284 | .20 | .655 | .004 | No* |
| **DeOxyHB** |  |  | |  |  |  |  |  |
| Channel 1,1 | 0.765 | 1,57 | | 0.765 | 1.77 | .188 | .030 | Yes |
| Channel 1,2 | 0.076 | 1,57 | | 0.076 | 0.30 | .584 | .005 | Yes |
| Channel 1,4 | 0.049 | 1,55 | | 0.049 | 0.18 | .672 | .003 | Yes |
| Channel 2,2 | 0.296 | 1,54 | | 0.296 | 0.79 | .379 | .014 | Yes |
| Channel 2,3 | 0.011 | 1,56 | | 0.011 | 0.04 | .834 | .001 | Yes |
| Channel 2,5 | 0.281 | 1,56 | | 0.281 | 1.68 | .200 | .029 | Yes |
| Channel 3,1 | 0.121 | 1,56 | | 0.121 | 0.49 | .487 | .009 | Yes |
| Channel 3,4 | 0.003 | 1,57 | | 0.003 | 0.01 | .919 | .000 | Yes |
| Channel 4,2 | 0.104 | 1,57 | | 0.104 | 0.53 | .469 | .009 | Yes |
| Channel 4,4 | 0.979 | 1,55 | | 0.979 | 1.22 | .274 | .022 | Yes |
| Channel 4,5 | 0.265 | 1,57 | | 0.265 | 0.74 | .394 | .013 | Yes |
| Channel 4,6 | 5.085 | 1,51 | | 5.085 | 4.45 | .040 | .080 | Yes |
| Channel 5,3 | 0.022 | 1,57 | | 0.022 | 0.21 | .650 | .004 | Yes |
| Channel 5,5 | 0.016 | 1,56 | | 0.016 | 0.07 | .798 | .001 | Yes |
| Channel 6,4 | 0.611 | 1,58 | | 0.611 | 1.05 | .310 | .018 | Yes |
| Channel 6,6 | 0.310 | 1,46 | | 0.310 | 0.19 | .664 | .004 | Yes |
| Channel 7,5 | 0.147 | 1,55 | | 0.147 | 0.80 | .376 | .014 | Yes |
| Channel 7,6 | 1.368 | 1,33.84 | | 1.368 | .92 | .343 | .018 | No* |

**Note:**

- SS = Sum of Squares
- df = Degrees of Freedom
- MS = Mean Square
- F = F-ratio
- p = p-value
- *Channels with a "No" under "Assumption of Homogeneity Met" had their results verified using Welch's F-statistic.

**Supplementary Table C4**

*fNIRS ANOVA Results for Go/No-Go Task (First 50 Seconds)*

| **Channel** | **SS** | **df** | **MS** | **F** | **p** | **ηp2** | **Assumption of Homogeneity Met** |
| --- | --- | --- | --- | --- | --- | --- | --- |
| **OxyHb** |  |  |  |  |  |  |  |
| Channel 1,1 | .016 | 1,53 | .016 | .016 | .901 | .000 | Yes |
| Channel 1,2 | .359 | 1,53 | .359 | .586 | .448 | .011 | Yes |
| Channel 1,4 | .340 | 1,51 | .340 | .954 | .333 | .018 | Yes |
| Channel 2,2 | .046 | 1,54 | .046 | .061 | .805 | .001 | Yes |
| Channel 2,3 | .155 | 1,52 | .155 | .193 | .662 | .004 | Yes |
| Channel 2,5 | .127 | 1,52 | .127 | .237 | .628 | .005 | Yes |
| Channel 3,1 | .092 | 1,51 | .092 | .242 | .625 | .005 | Yes |
| Channel 3,4 | .606 | 1,53 | .606 | 1.651 | .204 | .030 | Yes |
| Channel 4,2 | .193 | 1,51 | .193 | .382 | .539 | .007 | Yes |
| Channel 4,4 | .068 | 1,52 | .068 | .161 | .690 | .003 | Yes |
| Channel 4,5 | .427 | 1,53 | .427 | .995 | .323 | .018 | Yes |
| Channel 4,6 | .813 | 1,48 | .813 | 1.093 | .301 | .022 | Yes |
| Channel 5,3 | .011 | 1,53 | .011 | .018 | .893 | .000 | Yes |
| Channel 5,5 | 1.236 | 1,51 | 1.236 | 2.921 | .094 | .054 | Yes |
| Channel 6,4 | .020 | 1,52 | .020 | .052 | .821 | .001 | Yes |
| Channel 6,6 | .232 | 1,45 | .232 | .720 | .401 | .016 | Yes |
| Channel 7,5 | .040 | 1,52 | .040 | .091 | .764 | .002 | Yes |
| Channel 7,6 | 4.227 | 1,45 | 4.227 | 9.257 | **.004** | .171 | Yes |
| **DeOxyHB** |  |  |  |  |  |  |  |
| Channel 1,1 | .384 | 1,52 | .384 | 2.046 | .159 | .038 | Yes |
| Channel 1,2 | .021 | 1,54 | .021 | .255 | .616 | .005 | Yes |
| Channel 1,4 | .015 | 1,51 | .015 | .171 | .681 | .003 | Yes |
| Channel 2,2 | .060 | 1,54 | .060 | .486 | .489 | .009 | Yes |
| Channel 2,3 | .038 | 1,52 | .038 | .250 | .619 | .005 | Yes |
| Channel 2,5 | .017 | 1,52 | .017 | .232 | .632 | .004 | Yes |
| Channel 3,1 | .051 | 1,51 | .051 | .599 | .443 | .012 | Yes |
| Channel 3,4 | .305 | 1,53 | .305 | 1.321 | .256 | .024 | Yes |
| Channel 4,2 | .036 | 1,46.14 | .036 | .524 | .473 | .010 | No* |
| Channel 4,4 | .024 | 1,52 | .024 | .134 | .715 | .003 | Yes |
| Channel 4,5 | .217 | 1,53 | .217 | 1.204 | .277 | .022 | Yes |
| Channel 4,6 | .803 | 1,48 | .803 | 1.112 | .297 | .023 | Yes |
| Channel 5,3 | .002 | 1,53 | .002 | .016 | .901 | .000 | Yes |
| Channel 5,5 | .228 | 1,51 | .228 | 1.512 | .224 | .029 | Yes |
| Channel 6,4 | .000 | 1,52 | .000 | .000 | .986 | .000 | Yes |
| Channel 6,6 | .050 | 1,45 | .050 | .208 | .650 | .005 | Yes |
| Channel 7,5 | .059 | 1,52 | .059 | .184 | .670 | .004 | Yes |
| Channel 7,6 | 2.067 | 1,45 | 2.067 | 3.313 | .075 | .069 | Yes |

**Note:**

- SS = Sum of Squares
- df = Degrees of Freedom
- MS = Mean Square
- F = F-ratio
- p = p-value
- *Channels with a "No" under "Assumption of Homogeneity Met" had their results verified using Welch's F-statistic.

**Supplementary Table C6**

*fNIRS ANOVA Results for Cannabis Stroop Task (First 50 seconds)*

| **Channel** | **SS** | **df** | **MS** | **F** | **p** | **ηp2** | **Assumption of Homogeneity Met** |
| --- | --- | --- | --- | --- | --- | --- | --- |
| **OxyHb** |  |  |  |  |  |  |  |
| Channel 1,1 | 1.434 | 1,57 | 1.434 | 1.426 | .237 | .024 | Yes |
| Channel 1,2 | .038 | 1,57 | .038 | .037 | .849 | .001 | Yes |
| Channel 1,4 | .049 | 1,56 | .049 | .098 | .755 | .002 | Yes |
| Channel 2,2 | 1.532 | 1,56 | 1.532 | 1.387 | .244 | .024 | Yes |
| Channel 2,3 | .114 | 1,57 | .114 | .195 | .661 | .003 | Yes |
| Channel 2,5 | .054 | 1,57 | .054 | .104 | .749 | .002 | Yes |
| Channel 3,1 | .346 | 1,56 | .346 | .388 | .536 | .007 | Yes |
| Channel 3,4 | .140 | 1,57 | .140 | .169 | .682 | .003 | Yes |
| Channel 4,2 | .028 | 1,56 | .028 | .035 | .852 | .001 | Yes |
| Channel 4,4 | .199 | 1,56 | .199 | .269 | .606 | .005 | Yes |
| Channel 4,5 | .023 | 1,55 | .023 | .024 | .878 | .000 | Yes |
| Channel 4,6 | 2.384 | 1,53 | 2.384 | 1.171 | .284 | .022 | Yes |
| Channel 5,3 | .572 | 1,55 | .572 | .330 | .568 | .006 | Yes |
| Channel 5,5 | .712 | 1,55 | .712 | 1.249 | .269 | .022 | Yes |
| Channel 6,4 | 1.478 | 1,57 | 1.478 | 1.148 | .288 | .020 | Yes |
| Channel 6,6 | 1.731 | 1,48 | 1.731 | .266 | .609 | .006 | Yes |
| Channel 7,5 | 2.833 | 1,54 | 2.833 | 1.333 | .253 | .024 | Yes |
| Channel 7,6 | .992 | 1,46 | .992 | .647 | .425 | .014 | Yes |
| **DeOxyHB** |  |  |  |  |  |  |  |
| Channel 1,1 | .584 | 1,57 | .584 | 2.839 | .097 | .047 | Yes |
| Channel 1,2 | .001 | 1,57 | .001 | .003 | .953 | .000 | Yes |
| Channel 1,4 | .103 | 1,56 | .103 | 1.418 | .239 | .025 | Yes |
| Channel 2,2 | .061 | 1,56 | .061 | .403 | .528 | .007 | Yes |
| Channel 2,3 | .068 | 1,57 | .068 | .542 | .464 | .009 | Yes |
| Channel 2,5 | .046 | 1,57 | .046 | .367 | .547 | .006 | Yes |
| Channel 3,1 | .208 | 1,56 | .208 | 1.105 | .298 | .019 | Yes |
| Channel 3,4 | .077 | 1,57 | .077 | .501 | .482 | .009 | Yes |
| Channel 4,2 | .107 | 1,56 | .107 | .855 | .359 | .015 | Yes |
| Channel 4,4 | .006 | 1,56 | .006 | .032 | .859 | .001 | Yes |
| Channel 4,5 | .077 | 1,48.59 | .077 | .332 | .567 | .006 | No* |
| Channel 4,6 | .093 | 1,53 | .093 | .188 | .666 | .004 | Yes |
| Channel 5,3 | .434 | 1,55 | .434 | .617 | .435 | .011 | Yes |
| Channel 5,5 | .035 | 1,43.12 | .035 | .256 | .615 | .005 | No* |
| Channel 6,4 | 1.281 | 1,57 | 1.281 | 1.810 | .184 | .031 | Yes |
| Channel 6,6 | .040 | 1,37.58 | .040 | .051 | .822 | .001 | No* |
| Channel 7,5 | 1.099 | 1,54 | 1.099 | 1.457 | .233 | .026 | Yes |
| Channel 7,6 | .153 | 1,46 | .153 | .516 | .476 | .011 | Yes |

**Note:**

- SS = Sum of Squares
- df = Degrees of Freedom
- MS = Mean Square
- F = F-ratio
- p = p-value
- *Channels with a "No" under "Assumption of Homogeneity Met" had their results verified using Welch's F-statistic.

**Supplementary Table C7**

*fNIRS ANOVA Results for Stop-Signal Task (First 50 seconds)*

| **Channel** | **SS** | **df** | **MS** | **F** | **p** | **ηp2** | **Assumption of Homogeneity Met** |
| --- | --- | --- | --- | --- | --- | --- | --- |
| **OxyHb** |  |  |  |  |  |  |  |
| Channel 1,1 | .158 | 1,57 | .158 | .17 | .678 | .003 | Yes |
| Channel 1,2 | .494 | 1,56 | .494 | .62 | .433 | .011 | Yes |
| Channel 1,4 | .023 | 1,55 | .023 | .06 | .815 | .001 | Yes |
| Channel 2,2 | 1.849 | 1,56 | 1.849 | 2.21 | .143 | .038 | Yes |
| Channel 2,3 | .193 | 1,58 | .193 | .31 | .583 | .005 | Yes |
| Channel 2,5 | .857 | 1,58 | .857 | 1.49 | .228 | .025 | Yes |
| Channel 3,1 | .089 | 1,57 | .089 | .13 | .719 | .002 | Yes |
| Channel 3,4 | .016 | 1,58 | .016 | .03 | .875 | .000 | Yes |
| Channel 4,2 | .606 | 1,58 | .606 | 1.41 | .241 | .024 | Yes |
| Channel 4,4 | 4.012 | 1,56 | 4.012 | 4.97 | .030 | .082 | Yes |
| Channel 4,5 | .611 | 1,58 | .611 | .89 | .349 | .015 | Yes |
| Channel 4,6 | 10.010 | 1,57 | 10.010 | 2.75 | .103 | .046 | Yes |
| Channel 5,3 | .248 | 1,58 | .248 | .40 | .527 | .007 | Yes |
| Channel 5,5 | .136 | 1,57 | .136 | .29 | .591 | .005 | Yes |
| Channel 6,4 | .052 | 1,58 | .052 | .10 | .752 | .002 | Yes |
| Channel 6,6 | .286 | 1,40.14 | .286 | .24 | .626 | .005 | No* |
| Channel 7,5 | .052 | 1,57 | .052 | .12 | .733 | .002 | Yes |
| Channel 7,6 | .752 | 1,52 | .752 | .74 | .392 | .014 | Yes |
| **DeOxyHB** |  |  |  |  |  |  |  |
| Channel 11 | .113 | 1,57 | .113 | .51 | .480 |  | Yes |
| Channel 12 | .239 | 1,56 | .239 | 1.17 | .284 |  | Yes |
| Channel 14 | .000 | 1,55 | .000 | .00 | .995 |  | Yes |
| Channel 22 | .544 | 1,56 | .544 | 3.17 | .080 |  | Yes |
| Channel 23 | .231 | 1,58 | .231 | 2.13 | .149 |  | Yes |
| Channel 25 | .332 | 1,50.25 | .332 | 4.32 | .043 |  | No* |
| Channel 31 | .139 | 1,57 | .139 | 1.18 | .282 |  | Yes |
| Channel 34 | .009 | 1,58 | .009 | .05 | .829 |  | Yes |
| Channel 42 | .368 | 1,58 | .368 | 3.89 | .053 |  | Yes |
| Channel 44 | 1.227 | 1,56 | 1.227 | 3.82 | .056 |  | Yes |
| Channel 45 | .086 | 1,58 | .086 | .38 | .538 |  | Yes |
| Channel 46 | 7.417 | 1,57 | 7.417 | 2.26 | .138 |  | Yes |
| Channel 53 | .320 | 1,58 | .320 | 2.74 | .103 |  | Yes |
| Channel 55 | .001 | 1,57 | .001 | .00 | .953 |  | Yes |
| Channel 64 | .517 | 1,58 | .517 | 1.58 | .213 |  | Yes |
| Channel 66 | .182 | 1,51 | .182 | .25 | .616 |  | Yes |
| Channel 75 | .033 | 1,57 | .033 | .26 | .612 |  | Yes |
| Channel 76 | .205 | 1,52 | .205 | .30 | .588 |  | Yes |

**Note:**

- SS = Sum of Squares
- df = Degrees of Freedom
- MS = Mean Square
- F = F-ratio
- p = p-value
- *Channels with a "No" under "Assumption of Homogeneity Met" had their results verified using Welch's F-statistic.

**Appendix D – Behavioural Regression Models**

**Regression examining the effects of indices of cannabis use on Go/No-Go Errors**

Three participants were removed from all hierarchical linear regressions as their total lifetime cannabis dose scores, more than 3 standard deviations above the mean, were indicative of excessive and potentially problematic cannabis use. These extreme values were not representative of the target sample of regular cannabis users and likely to exert disproportionate leverage on parameter estimates, distorting the regression results.

A hierarchical linear regression was conducted to examine the effects of age, sex, AUDIT-C scores, total lifetime cannabis dose, age of cannabis use onset and last 30 Days frequency on Go/No-Go errors. Age, sex and AUDIT-C scores were added to step one of the model. Lifetime dose, age of onset and last 30 day’s frequency were added to step two. Variance Inflation Factors suggested that multicollinearity was not a concern in any of the regression models.

The overall regression model was non-significant and explained 11% of variance in Go/No-Go scores (R^2^ = .11, *F*(6,21)=.422, p=.856). In step one, age, sex and AUDIT-C scores did not have a significant effect on Go/No-Go Errors (R^2^-change=.06, F-change (3,24)=.542, p=.658). In step two, total lifetime cannabis dose, age of cannabis use onset and last 30 day’s frequency of use did not predict any significant variance in Go/No-Go scores (R^2^-change=.04., F-change (3,21) =.35, p=.792). None of the individual predictors were associated with Go/No-Go errors.

**Supplementary Table D1**

*Hierarchical Regression Model Predicting Go/No-Go Errors*

| Variable | Cumulative | | |  | Simultaneous | | |
| --- | --- | --- | --- | --- | --- | --- | --- |
|  | R^2^-change | F-change (df) | p |  | β | p |  |
| *Step one* | .06 | .54 (3,24) | .658 |  |  |  |  |
| Age |  |  |  |  | -.11 | .751 |  |
| Sex |  |  |  |  | .12 | .615 |  |
| AUDIT C |  |  |  |  | .30 | .236 |  |
| *Step two* | .04 | .34 (6,21) | .792 |  |  |  |  |
| Total Lifetime Dose (SJUs) |  |  |  |  | -.06 | .848 |  |
| Age of Onset |  |  |  |  | .15 | .686 |  |
| Last 30 Day’s Frequency |  |  |  |  | .19 | .487 |  |

**Regression examining the effects of Indices of cannabis use on Stroop Interference Score**

A hierarchical linear regression with the same structure as the previous models was used to assess the effects of cannabis use on Stroop Interference (Reaction time in ms).

The overall regression model was non-significant and explained 34% of variance in Stroop Interference scores (R^2^ = .34, F(6,21)=1.81, p=.146). In step one, age, sex and AUDIT-C scores did not predict any significant variance in Stroop Interference scores (R^2^-change=.16, F-change (3,24)=1.54, p=.229). In step two, total lifetime dose, age of cannabis use onset and last 30 day’s frequency of use did not predict any significant variance in Stroop Interference scores (R^2^-change=.18., F-change (3,21)=.1.90, p=.161). None of the individual predictors were associated with Stroop Interference scores.

**Supplementary Table D2**

*Hierarchical Regression Model Predicting Stroop Interference*

| Variable | Cumulative | | |  | Simultaneous | | |
| --- | --- | --- | --- | --- | --- | --- | --- |
|  | R^2^-change | F-change (df) | p |  | β | p |  |
| *Step one* | .16 | 1.54 (3,24) | .229 |  |  |  |  |
| Age |  |  |  |  | -.12 | .688 |  |
| Sex |  |  |  |  | .13 | .526 |  |
| AUDIT C |  |  |  |  | -.28 | .194 |  |
| *Step two* | .17 | 1.90 | .161 |  |  |  |  |
| Total Lifetime Dose (SJUs) |  |  |  |  | -.30 | .307 |  |
| Age of Onset |  |  |  |  | -.38 | .244 |  |
| Last 30 Days Frequency |  |  |  |  | .-.22 | .359 |  |

**Regression examining the effects of Indices of cannabis use on Stop Signal Reaction Time (Integration Method)**

A hierarchical linear regression with the same structure as the previous models was used to assess the effects of cannabis use on SSRT scores.

The overall regression model was non-significant and explained 21% of variance in SSRT scores (R^2^ = .21, F(6,21)=920, p=.500). In step one, age, sex and AUDIT-C scores did not predict any significant variance in SSRT scores (R^2^-change=.15, F-change (3,24)=1.41, p=.265). In step two, total lifetime dose, age of cannabis use onset and last 30 day’s frequency of use did not predict any significant variance in SSRT scores (R^2^ Change=.06., F Change (3,21)=.57, p=.674). None of the individual predictors were associated with SSRT.

**Supplementary Table D3**

*Hierarchical Regression Model Predicting SSRT*

| Variable | Cumulative | | |  | Simultaneous | | |
| --- | --- | --- | --- | --- | --- | --- | --- |
|  | R^2-^change | F-change (df) | p |  | Β | p |  |
| *Step one* | .15 | 1.41 (3,24) | .265 |  |  |  |  |
| Age |  |  |  |  | .08 | .808 |  |
| Sex |  |  |  |  | -.05 | .839 |  |
| AUDIT C |  |  |  |  | -.05 | .958 |  |
| *Step two* | .06 | .52 (3,21) | .674 |  |  |  |  |
| Total Lifetime Dose (SJUs) |  |  |  |  | .35 | .728 |  |
| Age of Onset |  |  |  |  | .41 | .256 |  |
| Last 30 Days Frequency |  |  |  |  | -.31 | .763 |  |

**Appendix E. Exploratory Regressions on fNIRS Data**

Exploratory hierarchical regressions were conducted within the cannabis user group to examine whether cannabis use characteristics—lifetime dose, 30-day frequency, and age of onset—predicted neural activation (oxyHb and deoxyHb) across 18 prefrontal fNIRS channels.

For each of the three tasks (Go/No-Go, Cannabis Stroop, Stop Signal), regressions were run separately for:

- The full task epoch, and
- The first 50 seconds, to assess potential attenuation of haemodynamic response.

This resulted in 216 total models (3 tasks × 2 time windows × 2 haemodynamic signals × 18 channels). Full SPSS outputs are provided as supplementary material.

A partial correction threshold of p < .01 was applied to both the overall model and individual predictors. In cases where the model was non-significant (p > .01), significant predictors were not interpreted further due to poor model fit and elevated false positive risk.

One model met the exploratory significance threshold: lifetime cannabis dose significantly predicted reduced oxyHb activation at Channel 7,6 during the Go/No-Go task (β = –.84, p = .001). However, this result is considered unreliable, as Channel 7,6 was the most frequently pruned due to poor signal quality—excluded in 13 (Go/No-Go), 12 (Stroop), and 11 (Stop Signal) participants. Positioned at the top of the array, this channel was particularly affected by hair thickness, limiting optode contact. The finding is reported for transparency but not interpreted further.

No other models met the p < .01 threshold.

**Appendix F** – NASA TLX Tables

**Supplementary Table F1**

*NASA-TLX Subscale Scores: Go/No-Go*

|  | Cannabis Users | Controls |
| --- | --- | --- |
|  |  |  |
| Mental Demand | 31.33 (±21.68) | 37.67 (±21.45) |
| Temporal Demand | 38.00 (±23.18) | 52.83 (±22.22) |
| Effort | 26.00 (±21.39) | 30.67 (±22.23) |
| Performance | 56.67 (±21.75) | 62.67 (±24.24) |
| Frustration | 24.50 (±24.08) | 39.17 (±25.43) |

**Supplementary Table F2**

*NASA-TLX Subscale Scores: Cannabis Stroop*

|  | Cannabis Users | Controls |
| --- | --- | --- |
| Mental Demand | 30.67 (±23.84) | 41.83 (±23.73) |
| Temporal Demand | 32.50 (±25.08) | 42.33 (±26.35) |
| Effort | 28.17 (±21.79) | 31.50 (±25.33) |
| Performance | 68.00 (±21.08) | 63.00 (±27.87) |
| Frustration | 14.33 (±16.23) | 28.67 (±24.67) |

**Supplementary Table F3**

*NASA-TLX Subscale Scores: Stop Signal*

|  | Cannabis Users | Controls |
| --- | --- | --- |
| Mental Demand | 59.00 (±23.98) | 63.17 (±17.98) |
| Temporal Demand | 56.83 (±23.80) | 56.67 (±22.26) |
| Effort | 56.67 (±23.90) | 59.67 (±20.76) |
| Performance | 41.17 (±12.98) | 40.33 (±21.81) |
| Frustration | 37.17 (±24.55) | 49.5 (±25.06) |

**References**

Bush, K., Kivlahan, D. R., McDonell, M. B., Fihn, S. D., Bradley, K. A., & Ambulatory Care Quality Improvement Project (ACQUIP. (1998). The AUDIT alcohol consumption questions (AUDIT-C): an effective brief screening test for problem drinking. *Archives of internal medicine*, *158*(16), 1789-1795.

Di Lorenzo, R., Pirazzoli, L., Blasi, A., Bulgarelli, C., Hakuno, Y., Minagawa, Y., & Brigadoi, S. (2019). Recommendations for motion correction of infant fNIRS data applicable to multiple data sets and acquisition systems. *NeuroImage*, *200*, 511-527. <https://doi.org/10.1016/j.neuroimage.2019.06.056>

EASYCAP GmbH. (n.d.). *EEG recording cap* [Equipment]. <https://www.easycap.de/>

Field, M. (2005). Cannabis ‘dependence’ and attentional bias for cannabis-related words. *Behavioural pharmacology*, *16*(5-6), 473-476.

Fisk, J. E., & Warr, P. (1996). Age-related impairment in associative learning: The role of anxiety, arousal and learning self-efficacy. *Personality and Individual Differences*, *21*(5), 675-686.

Hart, S. G., & Staveland, L. E. (1988). Development of NASA-TLX (Task Load Index): Results of empirical and theoretical research. In *Advances in psychology* (Vol. 52, pp. 139-183). North-Holland.

Houben, K., & Jansen, A. (2011). Training inhibitory control. A recipe for resisting sweet temptations. *Appetite*, *56*(2), 345-349.

Howard, J. D., & Kahnt, T. (2021). To be specific: The role of orbitofrontal cortex in signaling reward identity. *Behavioral neuroscience, 135*(2), 210.

Huppert, T., Diamond, S., Franceschini, M., Boas, D. (2009). HomER: a review of time-series analysis methods for near-infrared spectroscopy of the brain. *Applied optics 48*(10). <https://dx.doi.org/10.1364/ao.48.00d280>

Logan, G. D., & Cowan, W. B. (1984). On the ability to inhibit thought and action: A theory of an act of control. *Psychological review*, *91*(3), 295. <https://psycnet.apa.org/doi/10.1037/0033-295X.91.3.295>

The MathWorks Inc. (2022). MATLAB version: 9.13.0 (R2022b), Natick, Massachusetts: The MathWorks Inc. <https://www.mathworks.com>

Menon, V., & D’Esposito, M. (2022). The role of PFC networks in cognitive control and executive function. *Neuropsychopharmacology*, *47*(1), 90-103.

Macatee, R. J., Carr, M., Afshar, K., & Preston, T. J. (2021). Development and validation of a cannabis cue stimulus set. *Addictive Behaviors*, *112*, 106643.

Montgomery, C., Fisk, J. E., Newcombe, R., & Murphy, P. N. (2005). The differential effects of ecstasy/polydrug use on executive components: shifting, inhibition, updating and access to semantic memory. *Psychopharmacology*, *182*, 262-276.

Penrose, L., & Raven, J. C. (1936). Progressive matrices. *British Journal of Medical Psychology*.

Scholkmann, F., & Wolf, M. (2013). General equation for the differential pathlength factor of the frontal human head depending on wavelength and age. *Journal of biomedical optics*, *18*(10), 105004. <https://doi.org/10.1117/1.JBO.18.10.105004>

Williams, J. M. G., Mathews, A., & MacLeod, C. (1996). The emotional Stroop task and psychopathology. *Psychological bulletin*, *120*(1), 3.
